# A Chloroplast COR413 Protein From *Physcomitrella patens* Is Required for Growth Regulation Under High Light and ABA Responses

**DOI:** 10.3389/fpls.2020.00845

**Published:** 2020-06-19

**Authors:** Cecilia Ruibal, Alexandra Castro, Andrea L. Fleitas, Jorge Quezada, Gastón Quero, Sabina Vidal

**Affiliations:** ^1^Laboratorio de Biología Molecular Vegetal, Instituto de Química Biológica, Facultad de Ciencias, Universidad de la República, Montevideo, Uruguay; ^2^Unidad de Biotecnología Vegetal, Instituto de Biología Molecular y Biotecnología, Carrera de Biología – Facultad de Ciencias Puras y Naturales, Universidad Mayor de San Andrés, La Paz, Bolivia; ^3^Departamento de Biología Vegetal, Facultad de Agronomía, Universidad de la República, Montevideo, Uruguay

**Keywords:** *Physcomitrella*, COR413, light, dehydration, ABA

## Abstract

*COR413* genes belong to a poorly characterized group of plant-specific cold-regulated genes initially identified as part of the transcriptional activation machinery of plants during cold acclimation. They encode multispanning transmembrane proteins predicted to target the plasma membrane or the chloroplast inner membrane. Despite being ubiquitous throughout the plant kingdom, little is known about their biological function. In this study, we used reverse genetics to investigate the relevance of a predicted chloroplast localized COR413 protein (*PpCOR413im*) from the moss *Physcomitrella patens* in developmental and abiotic stress responses. Expression of *PpCOR413im* was strongly induced by abscisic acid (ABA) and by various environmental stimuli, including low temperature, hyperosmosis, salinity and high light. *In vivo* subcellular localization of PpCOR413im-GFP fusion protein revealed that this protein is localized in chloroplasts, confirming the *in silico* predictions. Loss-of-function mutants of *PpCOR413im* exhibited growth and developmental alterations such as growth retardation, reduced caulonema formation and hypersensitivity to ABA. Mutants also displayed altered photochemistry under various abiotic stresses, including dehydration and low temperature, and exhibited a dramatic growth inhibition upon exposure to high light. Disruption of *PpCOR413im* also caused altered chloroplast ultrastructure, increased ROS accumulation, and enhanced starch and sucrose levels under high light or after ABA treatment. In addition, loss of *PpCOR413im* affected both nuclear and chloroplast gene expression in response to ABA and high light, suggesting a role for this gene downstream of ABA in the regulation of growth and environmental stress responses. Developmental alterations exhibited by *PpCOR413im* knockout mutants had remarkable similarities to those exhibited by *hxk1*, a mutant lacking a major chloroplastic hexokinase, an enzyme involved in energy homeostasis. Based on these findings, we propose that PpCOR413im is involved in coordinating energy metabolism with ABA-mediated growth and developmental responses.

## Introduction

As sessile and autotrophic organisms, plants have a unique lifestyle that requires the ability to sense and adapt to changing environmental conditions by adjusting metabolism and growth.

Abiotic stress caused by unfavorable environmental conditions greatly disturbs plant growth and influences the geographical distribution of plants in nature. In agriculture, crop productivity is highly limited by major environmental factors such as water deficit, extreme temperatures and salinity. These stress factors, despite the nature of the primary environmental signal, can lead to similar secondary effects, including cellular dehydration, photosynthesis impairment, metabolic dysfunction and oxidative damage to membranes and other cellular components (Zhu, 2016). Abiotic stress often affects photosynthesis by disturbing the photosynthetic electron transport and generating reactive oxygen species (ROS) which can cause severe injury to chloroplasts (Niyogi, 1999; Apel and Hirt, 2004; Asada, 2006; Miller et al., 2010).

Plants respond to abiotic stress by inducing a number of mechanisms to prevent cell damage and to sustain or resume metabolic activity during or after stress. Many stress-response mechanisms are well conserved among all plants, but others have diverged during evolution and are expressed as important differences between plant species. Some of these differences derive from the presence or absence of specific anatomical adaptations to the terrestrial environment. For example, tracheophytes display adaptive traits that allow them to control water content by means of thick cuticles, stomata, vascular tissues and root systems. In contrast, bryophytes lack such adaptations and, in order to survive in the terrestrial environment, they must rely on efficient molecular/biochemical strategies to limit the stress-induced cell damage to a repairable level (Oliver et al., 2005).

Bryophytes, comprising mosses, liverworts and hornworts, are at the base of the evolution of land plants and as such, they can provide insight into the adaptive mechanisms that allowed the transition from water to land from their common ancestor with vascular plants (Kenrick, 2017; Rensing, 2018). Among the conserved adaptive mechanisms, is the synthesis and function of the plant hormone abscisic acid (ABA), which acts as a central regulator of water stress responses in all land plants (Cutler et al., 2010; Umezawa et al., 2010; Takezawa et al., 2011; Amagai et al., 2018; de Vries et al., 2018). Several environmental signals, including light and extreme temperatures, induce ABA accumulation in different plant species. However, dehydration and salinity are considered the most important factors controlling ABA accumulation (Xiong and Zhu, 2003).

In higher plants, ABA regulates many aspects of plant growth and development, such as seed development and maturation, germination and stomatal closure (Chater et al., 2011; Finkelstein, 2013). In addition, ABA is required for transcriptional activation of genes involved in responses to dehydration, low temperature and other stresses (Verslues and Bray, 2006; Nakashima et al., 2009). Activation of ABA responsive genes leads to the synthesis of proteins belonging to different families, including chloroplastic proteins involved in photosynthesis performance and ROS production and detoxification (Rook et al., 2006). Other genes regulated by ABA encode transcription factors, chaperones, enzymes for osmolyte biosynthesis, late embryogenesis abundant (LEA) proteins and cold regulated (COR) proteins (Bray, 1997; Thomashow, 1999; Finkelstein and Gibson, 2002; Vishwakarma et al., 2017). *COR* genes are named as such because they were initially identified in *Arabidopsis* as part of the transcriptional activation of plants during cold acclimation (Thomashow, 1999). Cold acclimation is a process based on exposure to low, non-freezing temperatures, that enables the acquisition of freezing tolerance in some plant species (Thomashow, 1999). This process has been largely conserved during plant evolution, and involves the accumulation COR proteins belonging to different families, including the LEA proteins (Thomashow et al., 1997; Thomashow, 1999; Hundertmark and Hincha, 2008; Nakayama et al., 2008; Thalhammer et al., 2010; Bhyan et al., 2012; Beike et al., 2015; Tan et al., 2017). The best characterized *COR* gene is *COR15A*, which accumulates in the chloroplast stroma and participate in freezing tolerance by protecting chloroplast membranes during stress (Steponkus et al., 1998; Thalhammer et al., 2014; Bremer et al., 2017).

A much less characterized group of *COR* genes belong to the COR413 family, encoding plant-specific multispanning transmembrane proteins of unknown function (Breton et al., 2003). COR413 proteins are grouped into two distinct subclasses according to their predicted intracellular localization: chloroplast COR413-TM/IM (thylakoid membrane/inner membrane) and plasma membrane COR413-PM (Breton et al., 2003). Although chloroplast COR413 proteins were originally predicted to localize on thylakoid membranes (Breton et al., 2003), more recently, *Arabidopsis* AtCOR413IM-1 and AtCOR413IM-2 were experimentally shown to target the inner membrane of these organelles (Okawa et al., 2008, 2014).

In the past years, *COR413* genes have been described in various plant species, including *Arabidopsis*, wheat, rice, sorghum, tomato, cotton, rape, *Xerophyta viscosa* and *Chrysanthemum* (Breton et al., 2003; Garwe et al., 2006; Wang et al., 2007; Okawa et al., 2008; Chen et al., 2011, 2014; Ma et al., 2017; Su et al., 2018). Accumulation of COR413 proteins is associated with the development of freezing tolerance in cereals (Breton et al., 2003; Colton-Gagnon et al., 2014). In addition, expression of *COR413* genes has been shown to be upregulated by ABA, low temperature or dehydration treatments in different plants (Machuka et al., 1999; Seki et al., 2002; Garwe et al., 2006; Cuming et al., 2007). Moreover, heterologous overexpression of genes encoding plasma membrane COR413 proteins, XvSAP1 or PsCOR413pm2, isolated from the desiccation tolerant plant *X. viscosa*, or *Phlox subulata* respectively, enhanced tolerance to salinity, osmotic stress, heat or low temperature in transgenic *Arabidopsis* (Garwe et al., 2006; Zhou et al., 2018). Similarly, a gene encoding a chloroplast COR413 from tomato (*SlCOR413IM1*) increased drought tolerance in tobacco plants (Ma et al., 2017) and overexpression of rice COR413-TM1 resulted in drought tolerance in this plant species (Zhang et al., 2017).

Recently, an *Arabidopsis* knockout (KO) mutant unable to accumulate AtCOR413-PM1 protein, was shown to be more sensitive to freezing stress than the wild type genotype (Zhang et al., 2017). Nevertheless, *Arabidopsis* KO mutants of genes encoding chloroplast variants of COR413, showed no substantial phenotypic differences with the wild type (Okawa et al., 2008). Thus, genetic evidence revealing the *in vivo* function of this type of genes in plants is still lacking.

The moss *Physcomitrella patens* is an important model for investigating gene function due to the remarkable simplicity of the system to perform targeted mutagenesis via homologous recombination (Kamisugi et al., 2006). As a representative ancestor of land plants, this moss is also an ideal model for studying the evolution of gene function in plants using reverse genetics (Cove et al., 1997).

Mosses, like most plants, show an alternation of generations, but unlike higher plants, the haploid gametophyte stage is dominant. This represents an additional advantage of *P. patens* as a model for reverse genetics, as mutant phenotypes are not masked by the presence of homologous alleles.

Like other plant species, growth and development of *P. patens* are regulated by environmental signals and phytohormones, such as auxin and cytokinin (Cove et al., 1978; Thelander et al., 2005). Moreover, ABA regulates growth and development by inhibiting plant growth and promoting the differentiation of brood cells (brachycytes), which serve as vegetative diaspores under adverse environmental conditions (Arif et al., 2019). Since the whole genome sequence of *P. patens* became available (Rensing et al., 2008), all the components of the core ABA signaling pathway known to operate in angiosperms have been identified in this plant species (Komatsu et al., 2009, 2013; Khandelwal et al., 2010; Chater et al., 2011; Takezawa et al., 2015). In addition, classical genetics approaches have led to the identification of a novel regulator of ABA responses in *P. patens*, the ANR protein kinase, which was not predictable from studies in flowering plants (Stevenson et al., 2016).

*Physcomitrella patens* has been proven to be an excellent system to study the function of genes involved in abiotic stress adaptations, since this plant is highly tolerant to a variety of environmental stress factors, including cold, freezing, salinity, dehydration and oxidative stress (Frank et al., 2005; Oliver et al., 2005; Oldenhof et al., 2006; Saavedra et al., 2006; Cuming et al., 2007; Charron and Quatrano, 2009; Koster et al., 2010; Pressel and Duckett, 2010; Stevenson et al., 2016; Tan et al., 2017).

In this study, we employed reverse genetics to characterize a novel ABA-induced *COR413* gene from *P. patens* (*PpCOR413im*), which was isolated from a subtractive library enriched in ABA upregulated genes (Ruibal et al., 2013). *PpCOR413im* encodes a chloroplast-targeted protein that is phylogenetically related to other members of the chloroplast subgroup of COR413 protein family from higher plants. We demonstrated that disruption of *PpCOR413im* affected plant growth and development, as well as responses to high light, osmotic stress, cold and dehydration. In addition, ABA mediated growth and developmental traits, as well as transcriptional responses were altered in the mutants, indicating a fundamental role for this type of proteins in plant adaptation to changing environmental conditions. Our study provides the first genetic evidence concerning the function of a chloroplast targeted COR413 protein in ABA-mediated developmental and stress responses.

## Materials and Methods

### Plant Material, Growth Conditions, and Treatments

*Physcomitrella patens* Grandsen strain (Schaefer et al., 1991) was used as wild type strain for all experiments, and as a background strain for gene targeted knockout genotypes. *hxk1* strain was kindly donated by Mattias Thelander. Plants were grown and maintained axenically on cellophane overlaid BCDAT medium as described by Ashton and Cove (1977). Protonemal cultures were generated by macerating moss colonies with ultra-turrax in sterile milliQ and plated onto cellophane-overlaid Petri dishes. For micropropagation, moss colonies or protonema were cut with a scalpel and plant fragments were transferred to fresh medium. Plants were grown at 22°C under a photoperiod of 16 h light, with a photon flux of 50 μmol m^–2^ sec^–1^, unless otherwise indicated. Three weeks-old colonies were used for most experiments.

*Arabidopsis thaliana* (Col-0) was used for stable transformation and confocal microscopy. For *in vitro* growth, seeds were surface sterilized for 10 min in 7% of bleach with 0.05% Tween-20, washed with sterile water, incubated at 4°C for 3 days and plated in Petri dishes with half strength MS medium (2.4 g L^–1^ Murashige and Skoog, 5 g L^–1^ sucrose, 0.5 g L^–1^ Monohydrate 2-ethanesulfonic acid and 10% agar). Plants were grown at 22°C with a photoperiod of 16/8 h and a photon flux of 120 μmol m^–2^ sec^–1^.

Treatment of *P. patens* with ABA, salicylic acid, NaCl and mannitol were performed by transferring plants onto plates with medium supplemented with these compounds at the indicated final concentrations. High light treatments were performed by exposing 20-days-old colonies to a photon flux of 200 μmol m^–2^ sec^–1^ (for medium high light: MHL) or to 350 μmol m^–2^ sec^–1^ (for high light: HL), or by continuous exposure to the indicated light conditions during 20 days. Heat stress and low temperature treatments were done by exposing 20-day-old colonies at 37°C or at 0–2°C, respectively, for the indicated times, with a photoperiod of 16/8 h and a photon flux of 50 μmol m^–2^ sec^–1^. Dehydration stress was done by transferring 7-day-old protonema to sterile Petri dishes overlaid with Whatman paper, and incubating them for 24 h inside a laminar flow. Osmotic stress was performed by growing colonies on BCDAT medium supplemented with mannitol, to a final concentration of 200 mM.

### Phylogeny and *in silico* Expression Analysis

Translated protein sequences from *P. patens*, *Arabidopsis* and wheat *COR413* genes were retrieved from Phytozome database and aligned with ClustalW (MEGA version 6) for phylogenetic analysis. The phylogenetic tree was constructed with the Neighbor joining method, using 1000 bootstrap replications.

*In silico* expression analysis of *P. patens COR413* genes were performed using microarray data from Genevestigator^1^ (Hruz et al., 2008) and from *Physcomitrella* eFP database^2^ (Ortiz-Ramírez et al., 2016).

### Construct Design

The construct for gene disruption of *PpCOR413im* was done using the vector pUBW302 (Saavedra et al., 2006), containing the *nptII* gene driven by the c 35S promoter and the 3′ UTR of the *ocs* gene. A 803 bp genomic fragment from the 5′ region of *PpCOR413im* (bases −314 to 488 from the start codon) was cloned upstream from the 35S promoter, whereas a 771 bp DNA fragment (bases 745 to 1511) from the 3′ genomic region was inserted downstream of *ocs* terminator. Primers used for PCR amplification of the 5′ genomic fragment were Fw: 5′-ggggtaccgtgcatgggtcagg-3′ and Rev 5′-aaagcttacccacagaccatactcgc-3′, containing *Kpn*I or *Hin*dIII restriction sites, respectively. The 3′ sequence of the gene was PCR amplified from genomic DNA using the primers Fw: 5′-cttggatccgcttcctgggtatggtgt-3′ and Rev: 5′-tgtctagagcaggagaggatggat-3′, containing *Bam*HI and *Xba*I restriction sites, respectively.

The construct for *in vivo* subcellular localization of PpCOR413im included the complete coding sequence of the intronless gene (without the stop codon), which was PCR amplified from genomic DNA using primers Fw: 5′-tgatggtaccgatggcctctcacatcgt-3′ and Rev:5′-atatctcgagtgcaacaccatacccaggaa-3′, containing *Kpn*I and *Xho*I restriction sites, respectively. The resulting PCR fragment was cloned into the pENTR2B vector (Gateway, Invitrogen), and recombined via LR clonase (Invitrogen) into the destination binary vector pK_7_FWG_2_ (Karimi et al., 2002). The resulting construct (35S:*PpCOR413im-GFP)* was introduced into *Agrobacterium tumefaciens* strain pGV3101/pMP90 by electroporation or used as such for protoplast transformation.

### *Arabidopsis* Transformation and Molecular Characterization of Transgenic Lines

The 35S:PpCOR413im-GFP construct was transformed into *Arabidopsis* by *Agrobacterium*-mediated floral dip transformation according to Clough and Bent (1998). Individual T2 kanamycin resistant plants were selected for molecular characterization. Expression of the transgene and presence of the PpCOR413im:GFP fusion protein was tested by RT-PCR. Plants were analyzed for *in vivo* intracellular localization of PpCOR413im:GFP.

### Generation and Molecular Characterization of *PpCOR413im* Knockout Mutants in *P. patens*

Preparation and transformation of *P. patens* protoplasts were done according to Schaefer et al., 1991. Thirty μg linearized DNA plasmid (*Knp*I digested) were used for PEG-mediated protoplast transformation. After two rounds of incubation with selection, alternated with non-selective conditions, resistant colonies were analyzed for the correct incorporation of the replacement construct at *PpCOR413im* locus by PCR amplification of genomic DNA using the primers: Fw-*a:* 5- cgatcgcgatggacaattctctct-3′, Rev-*b*: 5′-ctaactgcatcaagaacacagagaaag-3′, Fw-*c*: 5′-ctacccgtgatattgctgaagagc-3′ and Rev-*d*: 5′-cacctggaatctgtgaaaactaac-3′. To confirm the loss-of-function of the *PpCOR413im*, gene expression was analyzed by Northern blot using total RNA samples from wild type and mutant strains, from control or ABA (10 μM), treated during 24 h. Blotted samples were hybridized using the complete coding sequence of *PpCOR413im* labeled with [α^32^P]-dCTP, as a probe.

### *In vivo* Subcellular Localization of PpCOR413im-GFP

*Physcomitrella patens* protoplasts and transformation procedures were performed according to Schaefer et al., 1991. Fifteen μg of circular DNA plasmid (35S:PpCOR413im-GFP) were used for PEG-mediated protoplast transformation. Transgenic *Arabidopsis* leaves or transiently transformed protoplasts were analyzed for GFP signal using confocal laser scanning microscope Leica TCS-SP5 at 488 nm excitation. GFP fluorescence was measured at 503 to 515 nm (green) emission spectra, while chlorophyll autofluorescence was measured at 650–710 nm (red). All images were acquired using the same microscope settings.

### Northern Blot

RNA analysis from control or treated *P. patens* plants were performed as described in Ruibal et al., 2012. For probe preparation, the cDNA regions of the target genes were amplified with the following primers: *PpCOR413im* (Fw-5′-tgatggtaccgatggcctctcacatcgt-3′ and Rev-5′-cacagcggccgcgaaaattcaatcac-3′), *PsbA* (Q6YXN7) (Fw-5′-cttgctacatgggtcgtgagtg-3′ and Rev-5′-tgctgatacctaatgcagtgaacc-3′), *PsbD* (Q6YXN8) (Fw-5′-ttgtaggttggtctggtctattac-3′ and Rev-5′-aattcagggtcttcagctgcacga-3′). Probes were labeled using the MegaPrime DNA Labeling System kit (GE Healthcare, Amersham) and purified by G-25 columns from GE Healthcare, Amersham. Radioactive membranes were exposed on a cassette for 1 day and revealed in a photoimage.

### qRT-PCR

Total RNA samples from *P. patens* were isolated with RNeasy Plant Mini Kit according to manufacturer’s instructions (Qiagen, Germany). For cDNA synthesis, 2 μg of total RNA were reverse transcribed with QuantiNova Reverse Transcription (RT) Kit (Qiagen, Germany). To estimate amounts of cDNA templates of the selected genes, quantitative RT-PCR assays were performed using specific primers listed in Supplementary Table 2, designed by Primer3Plus (Untergasser et al., 2012). qPCR was performed in an *Applied Biosystems StepOne* real-time PCR system. Each 10 μL reactions contained 5 μL of SYBR Green PCR Master mix (2 X), 0.5 μM primers mix and 2 μL of template cDNA (1/10 dilution). The thermocycler was programmed to run for 5 min at 95°C, followed by 40 cycles of 15 s at 94°C, 30 s at 60°C. Transcript accumulation of each gene was normalized relative to the constitutively expressed *E3 ubiquitin ligase* (Le Bail et al., 2013). Amplification efficiencies of the different primer combinations were all > 90%. Relative expression was determined using the 2^–Δ^ ^Δ^ *^*Ct*^* method (Livak and Schmittgen, 2001). Each data point is the mean value of three biological replicates. Two technical replicates were used for each sample.

### Transmission Electron Microscopy

Wild type and *Δcor-5* mutants strains were grown in normal medium for 20 days (controls) and then transferred to high light conditions (350 μmol m^–2^ sec^–1^) for 48 h. Gametophytes were fixed overnight in 3.5% glutaraldehyde in phosphate buffer pH 7.2. Samples were washed 5 times in phosphate buffer for 10 min and incubated at 4°C overnight in phosphate buffer containing 1% OsO, and washed again 5 times for 10 min in phosphate buffer. Subsequently, samples were dehydrated for 20 min successively in ethanol 25%, 50%, 75%, 95% (twice), 100% (twice) and acetone (twice). The impregnation of the samples was carried out in acetone:araldite for 30 min in a ratio of 2:1, 60 min in a ratio of 1:1, 60 min in a 1:2 ratio, and finally in pure araldite overnight at 4°C. Samples were acclimatized at RT and blocks were made with fresh pure araldite and allowed to polymerize for 48 h at 60°C. Three hundred nm slices were prepared using a RMC, MT-X ultramicrotome, equipped with a glass blade. Sections were stained with 1% methylene blue and the fields from the ultrathin sections (50 nm), were selected, mounted on 200 mesh lattice copper grids and subsequently contrasted with 2% uranyl acetate and 1% lead citrate. The grids were visualized in a Jeol Transmission Electron Microscope, JEM 1010, operated at 100 kV. The images of interest were captured with a digital camera HAMAMATSU C4742-95, coupled to MET.

### Analysis of Plant Growth and Development

Plant growth was monitored by measuring the area of at least 32 colonies of each genotype grown directly on BCDAT, using ImageJ program. Dry weight was measured after incubation of individual plant colonies for 16 h at 80°C. For the determination of chlorophyll content, each plant was ground up in a mortar containing 5 mL of 80% (volume in volume, v/v) acetone and the homogenized plant material was filtered to remove cell debris. Total chlorophyll was calculated as chlorophyll *a* + chlorophyll *b* (mg g^–1^ fresh weight) using the following formula: Chl*a* mg.g^–1^ = [(12.7 × Abs663) – (2.6 × Abs645)] × mL acetone mg^–1^ fresh tissue; Chl*b* mg g^–1^ = [(22.9 × Abs645) – (4.68 × Abs663)] × mL acetone mg^–1^ fresh tissue. Chlorophyll content, fresh weight and dry weight of plants were determined in three independent experiments using 3 plates containing 6 colonies of each genotype and per plate and stress condition.

For morphological studies, colonies were grown for 20 days on ammonium tartrate-free BCD plates or in BCDAT plates without cellophane, with the addition of 1 μM 1-Naphthaleneacetic acid (NAA), 1 μM ABA, 0.15 M glucose or 200 mM mannitol. For visualization of protonemal filaments, moss colonies were stained for 1 min with 0.05% toluidine blue in citrate-citric acid buffer and rinsed in water to remove excess dye.

### Fv/Fm and NPQ Measurements

Protonemal cultures of WT and *Δcor-5* mutants were propagated using ultra-turrax and grown in the same 6-wells Petri dishes, under standard conditions for 7 days at 23°C. Treatments were carried out for 2 days with high light (350 μmol m^–2^ sec^–1^) and low temperatures (0–2°C). For dehydration treatments, protonemal tissues were dehydrated in a Petri dish with whatman paper in a laminar flow for 24 h. Controls corresponded to plants grown during 7 days with standard light (50 μmol m^–2^ sec^–1^) and temperature of 23°C, with a photoperiod of 16/8 h.

Chlorophyll fluorescence was measured in a Hansatech FSMII PAM fluorometer, using a saturating light at 6000 μmol m^–2^ sec^–1^ and actinic light of 830 μmol m^–2^ sec^–1^. Before the measurements, disks were dark adapted for 20 min at RT. The parameters Fv/Fm and non-photochemical quenching (NPQ) were calculated as (Fm - Fo)/Fm and (Fm - Fm′)/Fm′. Data are presented as mean ± standard deviation of three to six independent experiments.

### Starch and Sucrose Content

Starch and sucrose contents were determined according to Sánchez et al. (1998) and Brányiková et al. (2011), respectively, with minor modifications. For soluble sugar content, approximately 30 mg of fresh plant material from plant colonies, were grounded with liquid nitrogen. Two hundred and fifty μL of 80% ethanol were added to each tube, vortexed, incubated at 80°C for 20 min with shaking, and centrifuged at 15,000 × *g* for 5 min at 4°C. Supernatants were transferred to fresh pre-cooled eppendorf tubes and kept on ice. One hundred and fifty μL of 80% ethanol were added to the pellet, vortexed, incubated at 80°C for 20 min with shaking, and centrifuged at 15,000 × *g* for 5 min at 4°C. Supernatants from these tubes were pooled with the respective supernatants from the first step. Finally, 250 μL of 50% ethanol were added to the pellet, vortexed, incubated at 80°C for 20 min, and centrifuged at 15,000 × *g* for 5 min at 4°C. Once again, supernatants were pooled and the soluble carbohydrate content was spectrophotometrically determined using the Megazyme Sucrose/D-Fructose/D-Glucose Assay Kit (Megazyme Inc.) following the manufacturer’s instructions.

For starch content, 100 mg of fresh plant material were grounded with liquid nitrogen and extracted with 500 μL of 80% ethanol. 0.1 mL of a 1/5 dilution of the final extract were added to 1 ml of 0.2% anthrone solution [0.2 g of anthrone in 100 mL of 72% (v/v) H_2_SO_4_]. The mixtures were kept in a water bath at 100°C for 10 min and cooled to 20°C, and the Absorbance was measured at 625 nm. A standard curve using glucose was performed simultaneously. The starch contents were obtained by multiplying the interpolated values by 0.9.

For both measurements, wild type and *Δcor-5* mutant plants were grown under optimal conditions (Ctrl) for 20 days, and then transferred to 10 μM ABA supplemented plates, or exposed to light intensities of 200 μmol m^–2^ sec^–1^ (MHL) or 350 μmol m^–2^ sec^–1^ (HL) for 48 h.

### NBT Staining

*In situ* detection of superoxide was performed according to Jabs et al. (1996). *P. patens* colonies were grown for 20 days, transferred for 48 h to stress conditions and thereafter incubated with 1 mg mL^–1^ of nitro blue tetrazolium (NBT) in 10 mM potassium phosphate buffer pH 7.8, 10 nM NaN_3_. Colonies were incubated for 1 h prior to ethanol bleaching.

### Thylakoid Extraction and Large Pore Blue Native Gel Electrophoresis

Thylakoids from wild type or Δ*cor-5* strains were isolated exactly as described by Gerotto et al. (2019), except for the fact that, instead of protonema tissue, gametophytic colonies were used as plant material. Twenty days-old plant colonies, grown at standard light conditions (50 μmol m^–2^ sec^–1^) during the entire growth period (controls), or plants grown under identical conditions but transferred for 48 h to HL (350 μmol m^–2^ sec^–1^). Thylakoid extracts were solubilized with 1% (w/v) n-dodecyl β-D-maltoside (β-DM) at a final concentration of chlorophyll of 1 μg/μL and separated in large pore Blue Native-PAGE as described by Järvi et al. (2011). Gels were prepared using an acrylamide gradient of 3.5–12.5%(w/v) T and 3%(w/v) C in the separation gel, and 3%(w/v) T and 20% (w/v) C in the stacking gel, as described by Gerotto et al. (2019). Two independent experiments were performed.

### ABA Quantification

For ABA quantification, wild type and Δ*cor-5* mutant plants were grown under optimal conditions (Ctrl) or exposed during 48 h to MHL (200 μmol m^–2^ sec^–1^) or LT (0–2°C).

The extraction and purification of ABA was performed using the modified technique of Durgbanshi et al. (2005). As internal standards, 50 ng of the deuterated compound (2 H5) ABA was added. The separation was done by chromatography with an HPLC Alliance 2695 (Waters, Inc., CA, United States) equipped with a C18 reverse phase column (100 mm × 2.1 mm, 3-μm) with ionization by electrospray in negative form (ESI-) coupled with a triple-quadrupole mass spectrometer (Quattro Ultima pt; Micromass, Manchester, United Kingdom). Identification and quantification of ABA was carried out by injection of the samples in MRM mode (Multiple Reaction Monitoring), using the transitions for ABA 263 > 153 and for the deuterated form 268 > 159. The software used was MassLynx TM v. 4.1, Micromass (Manchester, United Kingdom). The results are expressed in pmol mg^–1^ of dry weight of plant tissue.

### Statistical Analysis

Statistically significant differences were determined based on the Student’s *t*-tests.

## Results

### *Physcomitrella patens* Genome Encodes a Single Chloroplast-Targeted COR413 Protein

A gene belonging to the *COR413* gene family was isolated from a previously constructed *P. patens*-subtracted cDNA library enriched in ABA-upregulated genes (Ruibal et al., 2013). The gene, designated here as *PpCOR413im*, is annotated in GenBank and in Phytozome databases with the accession numbers XP_024379790.1 and Pp3c7_22090V3.2, respectively. *PpCOR413im* encodes a 258-amino acid (27.02 kDa) deduced protein containing a single conserved multispanning transmembrane COR413 domain and a potential chloroplast transit peptide.

Using Phyre2 topology predictor program (Kelley et al., 2015), we analyzed the topology of PpCOR413im protein and compared the results with the predicted structure of AtCOR413IM1, an Arabidopsis ortholog for which there is significant structural information based on experimental data (Okawa et al., 2008, 2014). The results showed high degree of structural similarity between these two proteins, both of them containing 6 putative transmembrane helixes with N and C-termini facing the same side of the membrane (Supplementary Figure 1).

**FIGURE 1:**
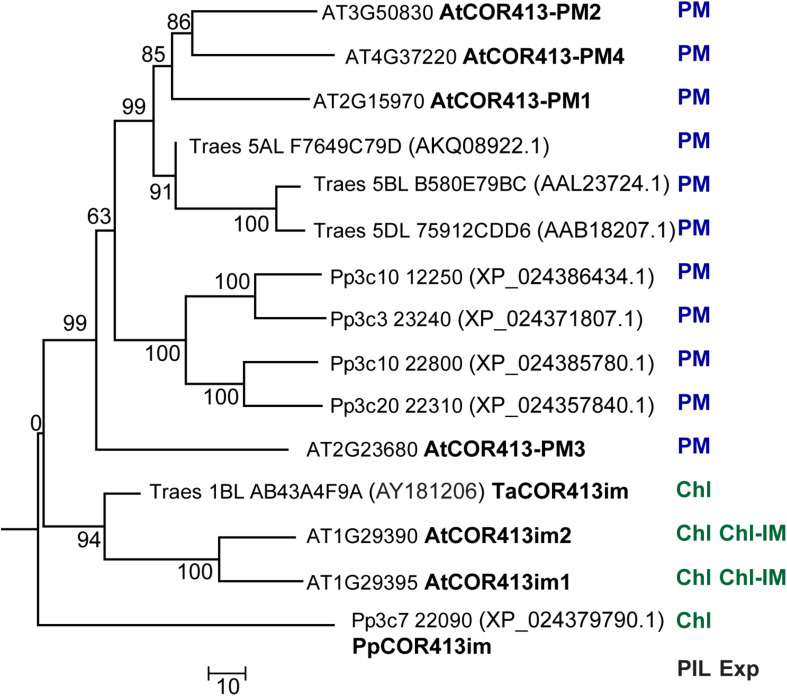
Phylogenetic relationships between COR413 proteins from *P. patens* (Pp) *Arabidopsis thaliana* (At), and *Triticum aestivum* (Ta) species. The phylogenetic tree was constructed by the Neighbor-joining method using ClustalW and MEGA6 software. For each sequence, gene ID (Phytozome database), GenBank accession number (in parenthesis, when it differs from Gene ID), and encoded protein name (when available), are shown to the right of the tree branches. PIL: Predicted intracellular localization of the encoded protein; Exp: Existing experimental evidence for the intracellular localization of the encoded protein; PM: Plasma Membrane; Chl: Chloroplast; IM: inner membrane. Bootstrap values are indicated in each node. Scale bar represents a distance of 10 substitutions per amino acid position.

A search for the presence of *COR413* genes in the *P. patens* genome using the conserved COR413 domain of *PpCOR413im* (residues 108–252 of the deduced protein), revealed the existence of four other genes encoding COR413 proteins (Phytozome ID Pp3c10 12250, Pp3c3 23240, Pp2c10 22800, and Pp3c20 22310). The phylogenetic relationship between the *COR413* gene family from *P. patens, Arabidopsis* and wheat, was analyzed using ClustalW sequence alignment (Thompson et al., 1997) followed by the neighbor-joining algorithm employing the MEGA 6 program (Tamura et al., 2011). PpCOR413im was placed in the same group together with chloroplast thylakoid membrane/inner membrane COR413 encoding genes from other species, while the other members of *P. patens* COR413 genes were grouped with the PM variants from other species (Figure 1).

The predicted subcellular localization of *P. patens* COR413 protein family was analyzed using GTP-Pp, a prediction tool trained with sequences from *P. patens* (Fuss et al., 2013). Analysis using GTP-Pp revealed that PpCOR413im was potentially targeted to chloroplasts but all other members of the protein family were likely to enter the secretory pathway and target the PM (Supplementary Table 1). Consistently, TargetP-2.0 analysis predicted the presence of a chloroplast transit peptide located within the first 71 amino acids of PpCOR413im. However, no consensus targeting signals were found in all other members of the protein family, which is in line with previous studies on other PM variants of COR413 proteins from different plant species (Breton et al., 2003).

**FIGURE 2:**
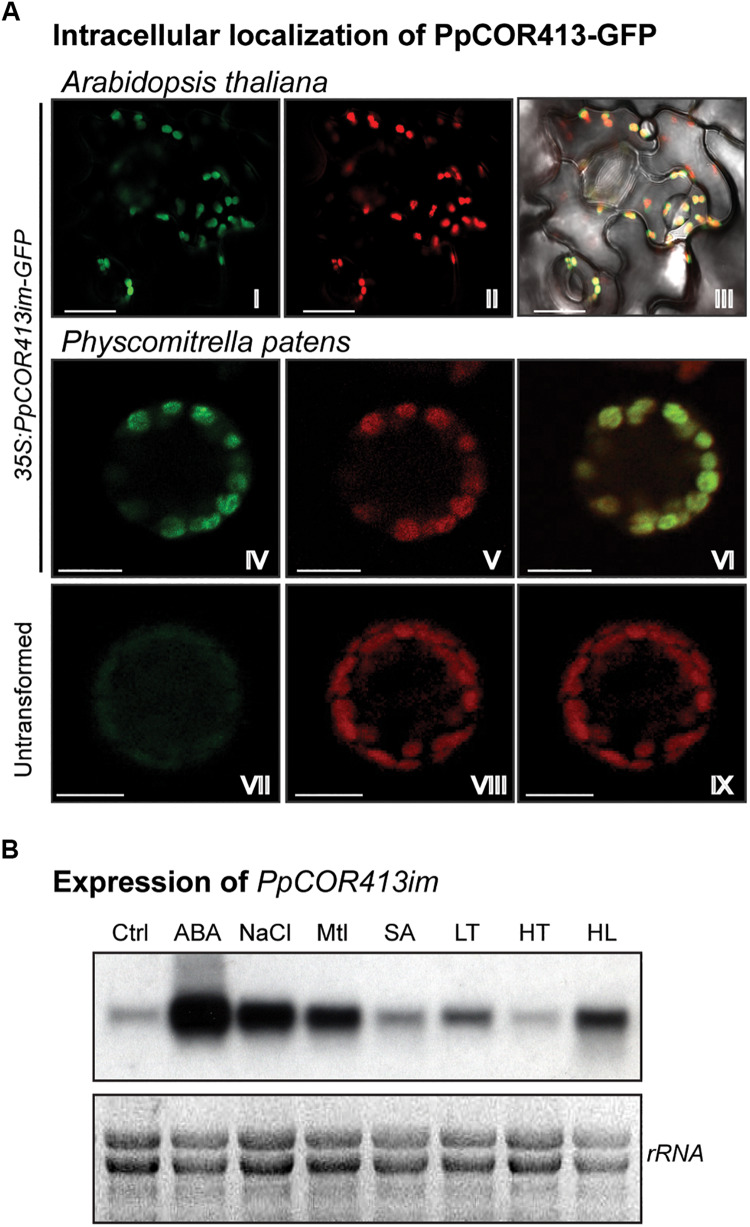
Subcellular localization, gene expression and predicted topology of PpCOR413im. **(A)** Subcellular localization of PpCOR413im. Confocal microscopy images of: Transgenic *Arabidopsis* lines overexpressing PpCOR413im-GFP fusion protein: I: GFP emission; II: chloroplast fluorescence; III: overlapping images. *P. patens* protoplasts transiently overexpressing PpCOR413im-GFP were photographed 48 h after transformation: IV: GFP signal; V: chloroplast fluorescence; VI: overlapping images. Untransformed P. patens protoplasts: VII: GFP signal; VIII: chloroplast fluorescence; IX: overlapping images. Scale bar, 10 μm **(B)** Northern blot analysis of *PpCOR413im* gene expression. Ten micrograms of total ARN were separated by electrophoresis, blotted onto nylon membranes and hybridized with [α−−^32^P]-dCTP labeled *PpCOR413im* full length sequence. Ctrl: untreated plants; ABA: treatment with 10 μM ABA; NaCl: 200 mM NaCl; Mtl: 400 mM mannitol (Mtl), SA: 1 mM salicylic acid; LT: low temperature (0–2°C); HT: high temperature (37°C), HL: high light (350 μmol m^–2^ sec^–1^). Treatments were done for 24 h. Ethidium bromide staining of rRNA was used to ensure equal loading of RNA samples.

To confirm the intracellular localization of PpCOR413im, we used *in planta* assays for visualization of PpCOR413im-GFP fusion proteins via transient expression in *P. patens* protoplasts as well as by the generation of stable transgenic *Arabidopsis* lines. For that purpose, we cloned the coding region of *PpCOR413im* fused in frame to GFP, and expressed the recombinant protein under the control of the constitutive CaMV 35S promoter (Figure 2A). Confocal microscopy was used to visualize *Arabidopsis* transgenic lines (Figure 2A I-III) or transformed *P. patens* protoplasts (Figure 2A IV-VI). In both systems, PpCOR413im-GFP localized in chloroplasts of *P. patens* or *Arabidopsis* cells, consistent with the predicted intracellular localization of the protein.

Based on the chloroplast localization of PpCOR413im and the structural similarities between this protein and AtCORIM-1, it is likely that PpCOR413im is an integral protein from the chloroplast inner membrane as shown for the Arabidopsis counterpart (Okawa et al., 2008, 2014).

### Expression of *PpCOR413im* Is Regulated During Development and Abiotic Stress

To gain insight into the biological function of *PpCOR413im* we assessed the expression profile of the gene in moss gametophyte colonies exposed to different hormonal treatments or stress conditions. RNA samples were extracted from plants treated for 24 h with 10 μM ABA or 1 mM salicylic acid (SA), or exposed to low temperature (LT: 0–2°C), high temperature (HT: 37°C), high light (HL: 350 μmol m^–2^ sec^–1^), osmotic stress (400 mM mannitol) or salinity (200 mM NaCl). Transcript accumulation of *PpCOR413im* was analyzed by Northern blot using the full-length cDNA sequence of the gene as a probe.

*PpCOR413im* exhibited a basal constitutive expression in gametophytes grown under control conditions, and was strongly induced by ABA, osmotic stress and salinity. In addition, the gene was upregulated in response to LT and HL treatments, while no increase in mRNA levels was observed in response to HT or SA treatments (Figure 2B).

*In silico* analysis of the expression profile of *PpCOR413im* and the four other *COR413* genes from *P. patens* were carried out using the microarray and RNA-seq based expression data from *P. patens* genes, available at Genevestigator (Hruz et al., 2008) and at the *P. patens* eFP database (Ortiz-Ramírez et al., 2016). Comparative expression profiles indicated that of the 25 assessed stimuli, only protoplasting, dehydration, rehydration and strong light treatments resulted in significant increases in *PpCOR413im* transcript levels (Supplementary Figure 2A). This analysis also indicated that the expression of different *PpCOR413* genes is independent of the predicted subcellular localization of the encoded proteins, as *PpCOR413im* exhibits almost identical expression profile as Pp3c20_22310, a gene encoding a deduced PM localized protein.

In addition, all *COR413* genes showed some level of expression under non-stress conditions during specific stages of the plant’s life cycle, but their expression pattern varied significantly between the genes (Supplementary Figure 2B). While most transcripts from PM variants of COR413 were abundant in sporophytes and gametophytes, *PpCOR413im* was found to be specifically expressed in the gametophytic phase of the plant’s life cycle (Supplementary Figures 2B,C).

Interestingly, *PpCOR413im* was significantly more expressed in caulonema than in chloronema, the last one being a far more chloroplast-rich type of tissue. This is intriguing since *PpCOR413im* is a chloroplast-targeted protein, and thus, is expected to exert its function in these organelles.

Promoter sequences up to 1.5 kbp upstream from the translation initiation site of the different *COR413* genes from *P. patens* were scanned using PLACE (Higo et al., 1999), PlantCARE (Lescot et al., 2002) and PlantPAN 3.0 (Chow et al., 2019) programs for the identification of *cis*-acting regulatory elements. Elements associated with stress and hormone responses were found in *PpCOR413im* and other members of the *COR413* gene family. The study revealed the presence of ABA response elements (ABRE and G-Box), light regulation and light stress (G-Box, Box-4, Box-II, GATA-motif), responses to dehydration (MBS, MYB and MYC), and auxin response (TGA) (Lam and Chua, 1989; Block et al., 1990; Ulmasov et al., 1995; Chen et al., 2002; Abe et al., 2003; Yamaguchi-Shinozaki and Shinozaki, 2006; Moabbi et al., 2012; Kaur et al., 2017). Regulatory elements associated with ABA, light regulation and dehydration stress were found in most gene promoters, with the exception of P3c10-22800, where only a few dehydration responsive elements were found (Supplementary Figure 2D). These results are consistent with the *in silico* analysis of gene expression, where P3c10-22800 was the only gene that showed no induction, or even down-regulation under excess light or dehydration and rehydration conditions (Supplementary Figure 2A). Interestingly, the TGA regulatory element was only present in *PpCOR413im* promoter region, suggesting a role for this gene in auxin responses.

### Generation of *PpCOR413im* Loss of Function Mutants

To investigate the physiological function of *PpCOR413im*, we generated gene-targeted mutants lacking a functional version of the gene. A gene-replacement construct was generated by replacing the second half of the single exon present in the gene, by the *nptII* gene, driven by the CaMV 35S promoter and *nos* terminator (Supplementary Figure 3A) and used for protoplast transformation.

Screening of a number of moss transformants lead to the identification of two independent KO lines (Δ*cor-1* and Δ*cor-5*). PCR analyses showed that both lines underwent homologous recombination at the 5′ end of *PpCOR413im* locus, while only Δ*cor-5* underwent homologous recombination at the 3′ end (Supplementary Figure 3B). Transcript accumulation of *PpCOR413im* was analyzed by Northern blot in ABA (50 μM)-treated WT and mutant lines, using the full-length cDNA fragment of *PpCOR413im* as hybridization probe. The results showed that neither of the two analyzed mutants showed expression of the target gene, indicating full disruption of *PpCOR413im* (Supplementary Figure 3C). Based on these results, both Δ*cor-1* and Δ*cor-5* lines were selected for phenotypic analysis.

### Disruption of *PpCOR413im* Alters Growth Rates Under High Light

**FIGURE 3:**
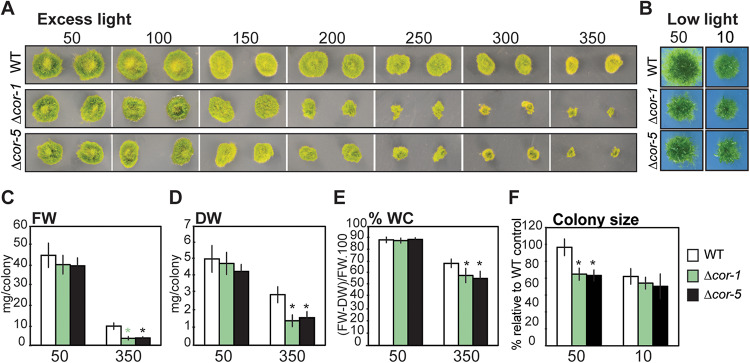
Effect of different light intensities on growth of wild type or *PpCOR413im* KO mutants. **(A)** Wild type (WT) or *PpCOR413im* KO mutants (*Δcor-1* and *Δcor-5*) were grown for 20 days under increasing light intensities (50–350 μmol m^–2^ sec^–1^). Pictures correspond to two representative colonies of each strain per treatment. **(B)** Pictures of representative colonies from WT or *Δcor-1* and *Δcor-5* lines grown during 20 days under standard light (50: 50 μmol.m^–2^.sec^–1^) or low light conditions (10: 10 μmol m^–2^ sec^–1^). **(C)** Fresh weight (FW), and **(D)** Dry weight (DW) per colony (mg) of control plants grown for 20 days under standard light conditions (50: 50 μmol m^–2^ sec^–1^), or under high light (350: 350 μmol m^–2^ sec^–1^) **(E)** Relative water content (%) per colony of plants grown as C and D. **(F)** Relative colony size of plants grown as B. The size of 32 individual colonies was quantified from photographic images using ImageJ software and the data is expressed as relative to WT controls: the values obtained for WT grown under optimal conditions were set as 100%. In all cases, the values shown are means from one representative technical replicate. Error bars indicate SD (*n* = 32). Three biological replicates were carried out. Significant differences of at least 0.05 confidence level between the WT and the KO lines are marked with an asterisk.

The intensity and quality of light are important factors regulating development, growth and responses to stress in plants. *P. patens* has relatively low light requirements for optimal growth (30–50 μmol m^–2^ sec^–1^), while stronger light intensities represent stressful conditions that have a negative impact on plant growth. This prompted us to analyze the KO lines for phenotypic alterations under different light intensities. To assess whether disruption of *PpCOR413im* affected plant growth under excess light conditions, plant colonies of KO mutants and WT were incubated at increasing light intensities (50 to 350 μmol m^–2^ sec^–1^) and growth rates were monitored after 20 days. Increasing light intensities resulted in a reduction of colony diameter when compared to control conditions, but this reduction was more significant for the KO mutants (Figure 3A). Exposure to light intensities of 200 μmol m^–2^ sec^–1^ or above, resulted in a significant reduction in growth rate of KO plants compared to the wild type. This reduction was particularly dramatic at the highest light intensity (HL: 350 μmol m^–2^ sec^–1^), as evidenced by the fresh weight and dry weight values of plant colonies (Figures 3C,D, respectively).

Interestingly, mutant plants grown under standard light conditions (50 μmol m^–2^ sec^–1^), showed a reduction of approximately 20% of the average colony diameters compared to the WT. However, no significant differences between the genotypes were observed when plants were grown at low light (10 μmol m^–2^ sec^–1^), indicating that the phenotypic alteration in growth rates is dependent on light intensity (Figures 3B,F).

As a consequence of prolonged exposure to HL, *in vitro* grown *P. patens* plants tend to experience certain level of dehydration. Interestingly, KO mutants exhibited significantly lower relative water contents than the WT when grown at HL (Figure 3E), suggesting that lack of *PpCOR413im* may also interfere with responses to plant dehydration.

In addition to HL, other abiotic stress factors causing cellular dehydration, such as osmotic stress, salinity or low temperature, induced high expression levels of *PpCOR413im* (Figure 2B). We therefore investigated the effect of loss of *PpCOR413im* in plant growth under osmotic and low temperature stresses by measuring the area of WT and KO colonies grown for 20 days on 200 mM mannitol supplemented plates (osmotic stress), or after 10 days exposure to ∼0–2°C. The presence of mannitol in the growth medium resulted in a larger reduction of colony sizes in the mutants (30%) than in the WT (10%), indicating that lack of *PpCOR413im* resulted in enhanced sensitivity to osmotic stress (Supplementary Figures 4A,B). However, no significant growth rate differences were observed between the WT and mutant lines after prolonged exposure to low temperature (Supplementary Figures 4C,D).

### Disruption of *PpCOR413im* Alters Caulonema Formation and Responses to ABA

In all conditions assayed, the two independent disruption lines were phenotypically indistinguishable from each other. Therefore, only Δ*cor-5* was included in further experiments of this study.

**FIGURE 4:**
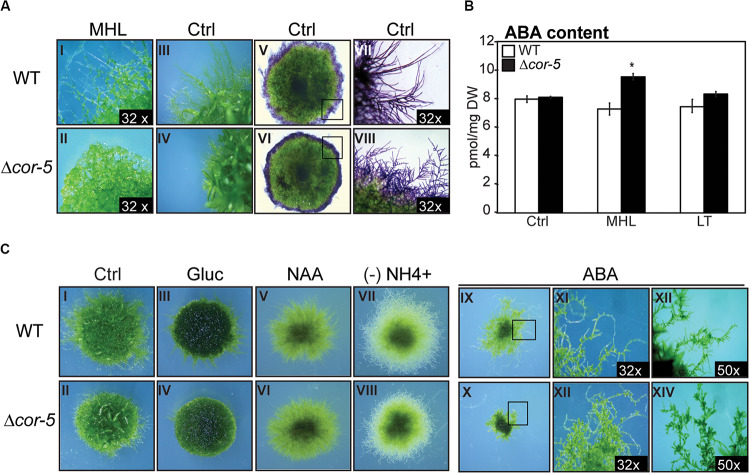
Morphological alterations of *PpCOR413im* KO mutant under different growth conditions. All plants were grown at 50 μmol m^–2^ sec^–1^, unless otherwise mentioned. Colonies from wild type (WT) and Δ*cor-5* mutants were grown under standard light conditions (50 μmol m^–2^ sec^–1^) unless otherwise indicated. **(A)** Stereoscopic images from protonemal tissue of 20 days-old WT and Δ*cor-5* colonies, grown under medium high light (MHL: 200 μmol m^–2^ sec^–1^) (I,II), or standard light conditions (Ctrl: 50 μmol m^–2^ sec^–1^) (III-VIII). Representative colonies of both genotypes, stained with toluidine blue are shown (V-VIII). Magnifications are 6.3 X or otherwise specified in the photograph. **(B)** WT and Δ*cor-5* were grown for 20 days under standard light conditions and exposed to MHL or low temperature (LT: 0-2°C) during 48 h. Samples from controls (Ctrl) or treated plants were used for quantification of ABA content, expressed in pmol mg^–1^ of dry weight (DW). The values shown are means from 4 biological replicates. Error bars indicate SD. Significant differences of at least 0.05 confidence level between the WT and KO lines are marked with an asterisk. **(C)** Stereoscopic images correspond to WT and Δ*cor-5* colonies grown for 20 days under standard light conditions. Ctrl: plants growing on BCDAT medium (I,II); Gluc: plants growing on BCDAT medium supplemented with 0.15 M glucose (III,IV); NAA: plants growing on BCDAT medium supplemented with 1 μM NAA (V,VI); (-) NH4^+^: plants growing on ammonium tartrate-free BCD medium (VII,VIII); ABA: plants growing on BCDAT medium supplemented with 1 μM ABA (IX-XIV). Magnifications are 6.3 X or otherwise specified in the photograph.

According to the RNA-seq and microarray available data, *PpCOR413im* is expressed primarily in the gametophores and caulonemal filaments (Supplementary Figures 2B,C). This prompted us to search for possible alterations of growth and developmental progression of Δ*cor-5* strain under different conditions. To evaluate responses to excess light, we selected a light intensity that was sufficient to induce a significant growth retardation phenotype in the mutants, and yet not high enough to cause apparent harm to the plants. This condition was defined as medium high light (MHL: 200 μmol m^–2^ sec^–1^). Stereoscopic images were taken from colonies of WT and Δ*cor-5* strains after 20 days of growth at control light conditions or at MHL (Figure 4A I-IV). In addition, WT and mutant colonies were stained with toluidine blue for proper visualization of the protonemal filaments (Figure 4A V-VIII). These experiments revealed important anatomical differences in the protonemal filaments of the KO mutant compared to the WT strain. First, caulonemal filaments were very poorly developed in Δ*cor-5.* Second, side-branching of protonemal filaments was significantly increased in the mutant compared to the WT, which is consistent with other reports showing modified filament branching phenotype in mutants of *P. patens* with altered caulonema differentiation (Thelander et al., 2005; Rawat et al., 2017).

The transition from chloronema to caulonema, as well as chloronemal branching, have been shown to be stimulated by auxin and high energy growth conditions (HL or glucose) (Ashton et al., 1979; Reski, 1998; Thelander et al., 2005, 2018). To further investigate caulonemal development in *PpCOR413im*-deficient mutants, we examined the effect of auxin treatment (1 μM naphthaleneacetic acid) or glucose supplementation (0.15 M) on the growth of WT and Δ*cor-5* strains (Figure 4C I-VI). Both treatments induced similar responses in WT and Δ*cor-5* genotypes. In fact, glucose supplementation reverted the reduced growth phenotype observed in Δ*cor-5* plants grown in the absence of glucose or under standard light intensity (Figure 4C III, IV). Thus, it is conceivable that the reduced growth observed in mutant plants is due to a reduction in energy availability, which can be compensated by adding glucose as an external carbon source. Protonemal growth is promoted by low nitrogen availability (Ashton and Cove, 1977; Jenkins and Cove, 1983; Thelander et al., 2005). To investigate whether *PpCOR413im* was required for the induction of protonemal growth by lack of nitrogen, Δ*cor-5* and WT colonies were cultivated in ammonium-free culture media and visualized after 20 days. No major phenotypic alterations were observed in the disruption mutants under these conditions (Figure 4C VII, VIII), indicating that *PpCOR413im* is not necessary for nitrogen-mediated growth responses.

Since *PpCOR413im* was strongly induced by ABA, and this hormone regulates protonemal development in *P. patens* (Thelander et al., 2005; Arif et al., 2019), we searched for possible alterations in ABA-mediated growth responses in *PpCOR413im* mutants. Treatment with 1 μM ABA severely attenuated plant growth in all genotypes, but growth inhibition was more dramatic in Δ*cor-5* than in the WT (Figure 4C IX, X). In addition, protonemal filaments of Δ*cor-5* were shorter than WT protonema, probably due to increased number of brood cells (Figure 4C XI-XIV). Taken together, these results suggest that disruption of *PpCOR413im* resulted in enhanced ABA sensitivity.

To examine whether growth defects of Δ*cor-5* mutants were due to elevated levels of endogenous ABA, we determined ABA content in WT and KO mutants grown under standard light conditions (Ctrl) or after 48 h exposure to MHL or LT. No significant differences in ABA levels were observed between the WT and KO plants when grown under standard light and temperature conditions, or after exposure to LT, suggesting that the observed developmental alterations were not due to changes in the endogenous levels of ABA (Figure 4B). However, treatment with MHL resulted in a slight but significant increase in ABA levels (30%) in Δ*cor-5* lines but not in the WT, suggesting that disruption of *PpCOR413im* altered both sensitivity to ABA and ABA accumulation in response to HL.

### *PpCOR413im* KO Mutants Exhibit Alterations in Photochemical Parameters Depending on the Environmental Stimuli

Extreme light intensities, cold and dehydration are among the many environmental factors that can compromise photosynthesis efficiency. To examine whether disruption of *PpCOR413im* affected photosynthesis performance, we used chlorophyll fluorescence to monitor the activity of photosystem II (PSII). Maximum PSII photochemical efficiencies (Fv/Fm) and other chlorophyll fluorescence parameters were measured in controls or after 48 h treatment of plants with HL, LT or after 24 h of dehydration. All treatments induced a decrease of Fv/Fm values in WT and Δ*cor-5* strains, but clear signs of photoinhibition were only observed after dehydration stress (Table 1). LT was the only treatment that induced significant differences between the genotypes, the KO strain showing slightly lower values of Fv/Fm than the WT.

**TABLE 1:** Fv/Fm.

	**Ctrl**	**HL**	**LT**	**DH**
WT	0.796 ± 0.015	0.717 ± 0.014	0.763 ± 0.007*	0.042 ± 0.001
Δ*cor-5*	0.798 ± 0.011	0.708 ± 0.029	0.735 ± 0.021*	0.046 ± 0.010

*PSII quantum yield (*F*v/*F*m) was analyzed in WT and Δ*cor-5* colonies grown under standard growth conditions (Ctrl) or after 24 h exposure to high light (HL), low temperature (LT) or dehydration (DH). Ctrl: (24°C and 50 μmoles m-2 s-1); HL: 24°C and 350 μmoles m-2 s-1; LT: 0–2°C and 50 μmoles m-2 s-1; and DH: 24 h dehydration in laminar flow. Values are reported as mean ± standard deviation (*n* = 6). Statistical significant differences between WT and Δ*cor-5* lines are marked with asterisks.*

Lowering Fv/Fm can arise from inactivation damage of PSII as a result from photooxidation (photoinhibition) and/or from inhibition of excess energy dissipation (Long et al., 1994; Tsonev et al., 2003; Demmig-Adams and Adams, 2006; Murchie and Lawson, 2013). One of the most important photoprotective mechanisms which helps plants to cope with over-excitation of the photosynthetic apparatus, is the non-photochemical quenching of chlorophyll *a* (NPQ). In this process, the excess chlorophyll excitation energy is dissipated by heat, preventing the formation of free radicals and thus mitigating photodamage (Kasakima et al., 2011). Consequently, mutants impaired in NPQ induction have shown to be more sensitive to environmental stress (Cousins et al., 2002; Dall’Osto et al., 2007; Allorent et al., 2013; Neto et al., 2014; Zhao et al., 2017).

**FIGURE 5:**
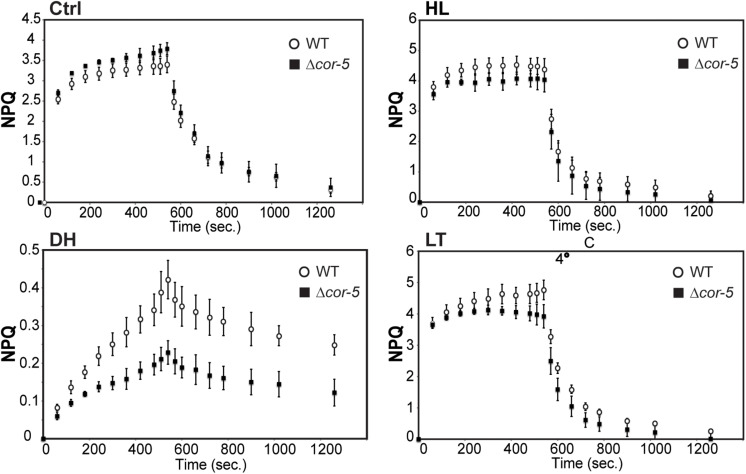
Non-photochemical quenching (NPQ) of *PpCOR413im* KO mutants and the WT. NPQ induction and relaxation of 7-days-old protonemal tissue from WT or Δ*cor-5*, grown under optimal conditions (Ctrl: 50 μmol m^–2^ sec^–1^), or exposed for 48 h to high light (HL: 350 μmol m^–2^ sec^–1^) or low temperature (LT: 0–2°C). Dehydration treatment was done for 24 h in the hood (DH). WT (white circles), *Δcor-5* plants (black squares). Data are presented as mean ± standard deviation of six independent experiments.

The ability of WT and Δ*cor-5* strains to induce NPQ was evaluated in pulse fluorometry time courses following illumination of dark-adapted plants with saturating light (Figure 5). NPQ levels were analyzed following 48 h exposure to HL or LT, or after 24 h dehydration treatment. NPQ maximum values in Δ*cor-5* were 8%, 18% or 46% lower than in the WT, for HL, LT or dehydration treatments, respectively, indicating that under all assessed stress conditions, WT plants underwent stronger light induced fluorescence quenching compared to the mutant lacking *PpCOR413im*. This indicates that Δ*cor-5* has reduced ability to induce photoprotective measures to cope with the excess of energy generated as a consequence of the stress-induced over-excitation of the photosynthetic apparatus. Interestingly, compared to the WT, Δ*cor-5* strain was only marginally affected in NPQ values under HL, but particularly altered in response to dehydration, the treatment that produced the highest level of photoinhibition. Nevertheless, the most noteworthy phenotypic differences between Δ*cor-5* and WT strains were observed under excess light conditions, where *PpCOR413im* KO mutants exhibited a marked increase in growth inhibition. The fact that photochemical parameters were only marginally altered *PpCOR413im* KO mutants after exposure to HL conditions, suggests that this protein plays distinct roles in light-mediated growth regulation and in dehydration or low temperature adaptive responses.

Taking into account that the biological roles of plant *COR413* genes have been predominantly studied in the context of cold or drought stress conditions (Breton et al., 2003; Garwe et al., 2006; Colton-Gagnon et al., 2014; Ma et al., 2017; Zhang et al., 2017; Zhou et al., 2018), in the present work we focused on deepen knowledge of the function of PpCOR413im in ABA and light responses.

### Disruption of *PpCOR413im* Alters Chloroplast ROS Accumulation and Expression of Photosynthetic Genes

Excess light and other environmental factors can induce the accumulation of chloroplastic ROS, such as singlet oxygen (^1^O_2_), superoxide anion radicals (O_2_^–^), hydrogen peroxide (H_2_O_2_), and hydroxyl radicals (Niyogi, 1999; Dietz et al., 2016). In addition, ROS production can be stimulated by ABA and act as secondary signals to regulate growth and transcriptional responses to stress.

**FIGURE 6:**
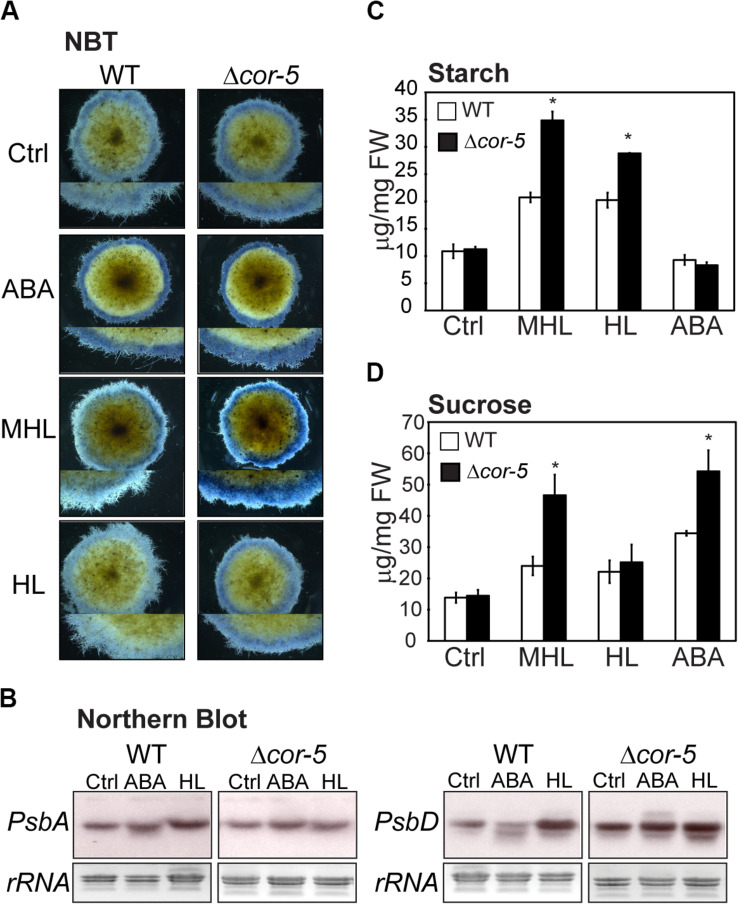
Stress-induced biochemical and molecular responses of WT and *PpCOR413im* KO mutants. **(A)** *In situ* determination of O_2_^–^ by NBT staining of *P. patens* WT or Δ*cor-5* colonies. Plants were allowed to grow for 20 days under optimal light and temperature conditions and thereafter were transferred to 10 μM ABA containing plates or exposed for 48 h to low temperature (LT: 0–2°C), medium high light (MHL: 200 μmol m^–2^ sec^–1^) or high light (HL: 350 μmol m^–2^ sec^–1^). Stereoscopic images from representative moss colonies of a pool of 6 individual colonies per genotype and per treatment are shown. Magnifications are 6.3 X **(B)** Northern blot analysis of photosynthetic genes of WT and *Δ-cor5* strains. Total RNA was isolated from control plants (Ctrl) or plants treated for 24 h with ABA (10 μM), or exposed to high light (HL: 350 μmol m^–2^ sec^–1^), or low temperature (LT: 0–2°C). The full-length cDNA sequences of *PsbA* and *PsbD* were labeled with [α−−^32^P]-dCTP and used as hybridization probes. Ethidium bromide staining of rRNA was used to ensure equal loading of RNA samples. **(C,D)** Effect of excess light intensity and ABA treatment on starch and sucrose accumulation of WT and Δ*cor-5* strains. Plants were grown and treated as A, and exposed for 48 h to MHL, HL or transferred 10 μM ABA containing plates for determination of starch content **(C)** or sucrose content **(D)**. Data are expressed as μg per mg of fresh weight (FW). Values shown are means from one representative technical replicate. Three biological replicates were carried out. Error bars indicate SD (*n* = 5). Significant differences of at least 0.05 confidence level between the WT and the KO lines are marked with an asterisk.

To assess whether high light and ABA hypersensitivity observed in Δ*cor-5* mutants, were accompanied by enhanced ROS accumulation, nitro blue tetrazolium (NBT) staining was used for histochemical detection of O_2_^–^ in plants grown under standard conditions (controls), and 48 treatment with ABA (10 μM), or exposure to two different excess light conditions: medium high light (MHL: 200 μmol m^–2^ sec^–1^) or high light (HL 350 μmol m^–2^ sec^–1^). Excess light treatments, particularly MHL, induced higher accumulation of O_2_^–^ in the protonemal filaments of Δ*cor-5* than in WT filaments tissues, suggesting a reduced ability to suppress stress-induced ROS accumulation in chloroplasts of mutant lines (Figure 6A).

Regulation of chloroplast gene expression is crucial for photosynthesis and development. Transcription and mRNA stability of chloroplast genes are modulated by developmental cues, environmental factors, ROS and hormones (reviewed in Börner et al., 2015). Many abiotic stress factors, including drought and high light, induce chloroplast-nucleus communication to activate adaptive responses (Chan et al., 2016; de Vries and Archibald, 2018), and ABA has been shown to be involved in this process (Holzinger and Becker, 2015; Zhao et al., 2019). For example, in higher plants, ABA represses the transcription of many plastid genes (Yamburenko et al., 2013, 2015). Moreover, redox alterations in chloroplasts, triggered by altered light conditions, influence the expression of genes encoding core proteins of PSI and PSII (Pfannschmidt et al., 1999; Kollist et al., 2019). In *P. patens*, transcription of *PsbA* and *PsbD* genes, encoding D1 and D2 core components of PSII, has been shown to be induced by HL conditions (Ichikawa et al., 2008).

In order to assess whether loss of *PpCOR413im* altered mRNA accumulation of *PsbA* and *PsbD* in response to HL or ABA, we used Northern blot hybridization to monitor transcript accumulation of these genes in WT and Δ*cor-5* strains. Plants were treated for 24 h with 10 μM ABA, or exposed to HL, and total RNA samples were hybridized with *PsbA* or *PsbD* cDNA probes. We found that HL treatment resulted in a modest induction of the expression of *PsbA* and *PsbD* in the WT, whereas KO plants exhibited no differences in transcript accumulation between HL treatment and controls (Figure 6B). Compared to the WT, Δ*cor-5* plants showed significant higher expression level of *PsbD* in controls, suggesting that Δ*cor-5* was altered in the steady state levels of *PsbD* transcript accumulation. Interestingly, ABA inhibited the expression of *PsbD* in WT plants, in contrast to the reported ABA-induction of *PsbD* gene in barley and *Arabidopsis* (Yamburenko et al., 2013, 2015). However, ABA treatment of Δ*cor-5* did not result in a reduction in *PsbD* mRNA levels, suggesting that *PpCOR413im* is required for proper ABA-regulation of this gene. Interestingly, gene expression analysis using RT-PCR, showed no significant differences between the WT and KO mutants in the accumulation of transcripts of other photosynthetic genes (*PsbS*, *LhcsR1* and *LhcsR2)*, encoding light harvesting antenna subunits of photosystems, which are required for photoprotection (Supplementary Figure 5).

### ABA and High Light-Induced Changes in Sugar Metabolism Are Altered in *PpCOR413im* KO Mutants

Sugar metabolism can be reorganized in response to abiotic stresses to maintain carbohydrate balance, prevent energy stress and thus, contribute to plant fitness (Smith and Stitt, 2007; Stitt and Zeeman, 2012; Thalmann and Santelia, 2017). In addition, sugars can act as signaling molecules, and interact with the ABA-dependent pathways to activate downstream stress responses (Rook et al., 2006). In *P. patens*, salinity, LT and ABA treatments were shown to increase the levels of soluble sugars, and decrease starch by accelerating starch degradation (Nagao et al., 2005; Gao et al., 2016).

To assess whether disruption of *PpCOR413im* altered sugar content and composition, sucrose and starch were quantified in WT or *Δcor-5* in controls or after 48 h exposure to MHL or HL intensities, as well as after treatment with 10 μM ABA. Our results showed that Δ*cor-5* accumulated higher levels of starch than the WT in response to excess light treatments (Figure 6C). Additionally, while all treatments induced sucrose accumulation in both plant strains, sucrose content reached significantly higher levels in Δ*cor-5* after treatments with MHL or ABA (Figure 6D). Thus, excess light -induced sucrose accumulation did not correlate with a reduction in starch concentration, suggesting that under these conditions, accumulated sucrose may originate from a different source than from starch degradation.

### Disruption of *PpCOR413im* Altered Nuclear Gene Expression in Response to ABA and High Light

To further understand the molecular basis for light stress and ABA hypersensitivity in *PpCOR413im* mutant plants, we monitored the expression pattern of several genes from *P. patens* involved in: ABA biosynthesis (*NCED*), signaling (*PP2C*), and ABA and stress responsive genes. NCED (9-*cis*-epoxycarotenoid dioxygenase) is the rate-limiting enzyme in ABA biosynthesis which catalyzes the conversion of violaxanthin to neoxanthin in the chloroplast (Nambara and Marion-Poll, 2005). *PP2C* encodes a member of the protein phosphatase type 2C family of proteins that act as negative regulators of ABA signaling (Fujii et al., 2009; Santiago et al., 2012; Soon et al., 2012). *TSPO* encodes a multi stress regulator membrane protein that is rapidly induced by ABA and a variety of abiotic stresses (Lindemann et al., 2004; Guillaumot et al., 2009). *DHNA* encodes a dehydrin protein that has been shown to confer tolerance to osmotic stress and salinity in *P. patens* (Saavedra et al., 2006). Finally, *SUS* encodes a sucrose synthase with participates in sucrose metabolism and starch biosynthesis (Geigenberger and Stitt, 1993; Angeles-Núñez and Tiessen, 2010).

**FIGURE 7:**
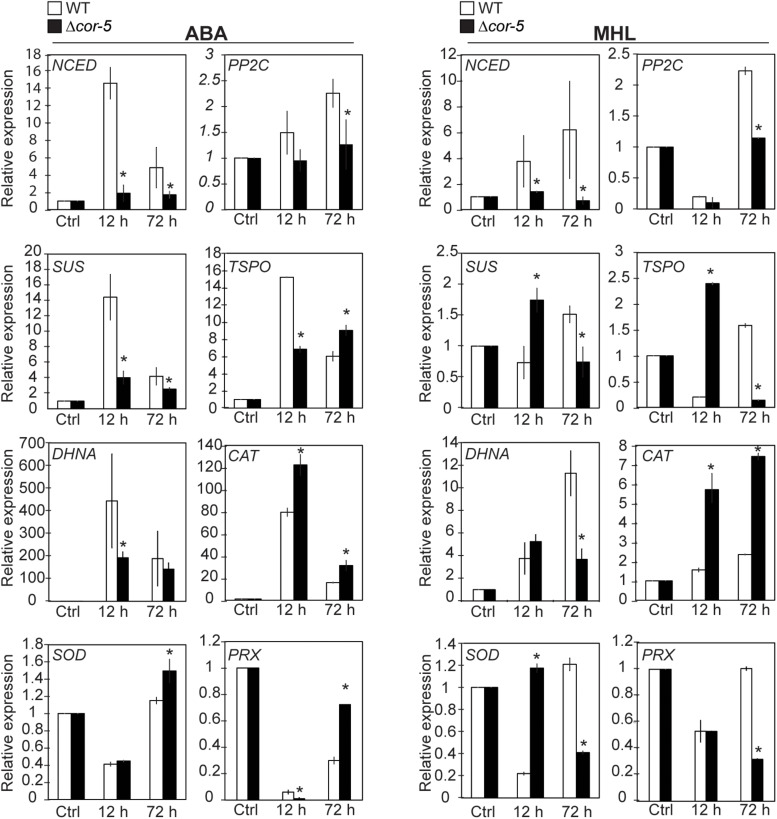
Expression of ABA responsive genes and antioxidant enzyme genes in WT and *PpCOR413im* KO mutant. Twenty-day-old colonies of the WT and Δ*cor-5* mutant were treated with ABA (10 μM) or exposed to medium high light (MHL: 200 μmol m^–2^ sec^–1^), and total RNA samples were isolated after 12 or 72 h after treatments, and from untreated control plants (Ctrl). Quantitative RT-PCR was used to analyze the expression of *NCED* (Pp3c16_17210), *PP2C* (Pp3c14_25570), *SUS* (Pp3c5_19770), *TsPO* (Pp3c2_17540), *DHNA* (Pp3c12_900), *CAT* (Pp3c19_6540), *SOD* (Pp3c9_24840), and *PRX* (Pp3c4_14530). The *Elongation factor 1*α gene (Pp3c2_6770) was used as internal control. Expression levels are reported as relative to control samples, and values represent means ± SE of three biological replicates. Significant differences of at least 0.05 confidence level between the WT and Δ*cor-5* strains are marked with an asterisk. Specific primers used for PCR amplification are shown in Supplementary Table 3.

Colonies from WT and *Δcor-5* strains were treated with 10 μM ABA or exposed to MHL and total RNA samples were extracted after 12 h or 72 h of treatments for subsequent analysis of gene expression by qRT-PCR (Figure 7). All genes were induced by ABA or by MHL in WT and Δ*cor-5*, but differences between the genotypes were observed in terms of timing or induction level. WT plants consistently showed a significantly stronger transcriptional response of *NCED* and *PP2C* genes. ABA treatment resulted in the early and sustained upregulation of *PP2C* only in the WT genotype, while no or low- induction levels were detected in Δ*cor-5*. The expression of *NCED* was rapidly induced in WT plants, reaching maximal induction levels at 12 h (15-fold) after ABA treatment, or at 72 h (6-fold induction) upon exposure to MHL. In contrast, KO mutants failed to exhibit a significant induction of *NCED* in response to ABA or MHL. In this case, maximum induction levels (2-fold induction over the controls) were observed at 12 h after ABA treatment.

Similarly, the induction of *SUS, DHNA* and *TSPO* genes by ABA was higher in the WT than in the KO, particularly at early time points. In the WT, the ABA-induced gene expression peaked at 12 h, but the fold increase declined with time, while in KO plants, the induction of these genes by ABA was significantly lower than the WT, particularly at early time points. On the contrary, the expression of *SUS* and *TSPO* in response to MHL was higher in the KO than in the WT at early time points but showed an opposite expression pattern after long-term exposure to MHL. In the case of *DHNA*, genotypic differences in gene expression were only detected after prolonged exposure to MHL, with a higher induction level in the WT than in the KO strain.

The fact that *PpCOR413im* KO strains exhibited ABA hypersensitivity and at the same time failed to induced genes involved in ABA synthesis or signaling in response to ABA treatment, suggest that *PpCOR413im* may interfere with the feedback regulatory circuit of ABA accumulation. Particularly relevant to this idea is the fact that members of the *PP2C* gene family have been suggested to be involved in this ABA self-regulatory loop (Hugouvieux et al., 2001). In addition, miss regulation of ABA accumulation and signaling may in turn interfere with stress regulation of response genes, as both temporal expression and induction levels of *SUS, DHNA* and *TPSO* were altered in the mutant. Taking together, these results suggest that *PpCOR413im* is required for proper modulation of ABA gene expression and ABA homeostasis.

In addition to the observed alterations in ABA responses, disruption of *PpCOR413im* resulted in accumulation of ROS under stress conditions, particularly altered under light conditions such as MHL and in some degree HL. Excess ROS produced as a result of chloroplast stress can diffuse to the cytoplasm and affect nuclear expression of enzymes involved in ROS detoxification (Nott et al., 2006).

To monitor ROS metabolism, we examined the expression of antioxidant enzyme genes, encoding a Cu-Zn chloroplast superoxide dismutase (SOD), peroxiredoxin (PRX), and a catalase (CAT). Detoxification of superoxide by SOD is an important mechanism to regulate ROS accumulation in chloroplasts (Higashi et al., 2015), while PRX and CAT are crucial for eliminating hydrogen peroxide in the chloroplast and in the cytosol, respectively (reviewed in Pospíšil, 2016).

We found that ABA and MHL induced the expression of *CAT* to a higher extent in Δ*cor-5* than in the WT. Expression of *SOD* was either repressed or slightly induced after 72 h of ABA treatment, but this induction was specifically observed in the KO strain. Nevertheless MHL treatment resulted in similar inhibition levels of *SOD* gene expression in both genotypes, but in the KO strain, this response was delayed in time compared to the WT. Finally, expression of *PRX* was repressed by ABA and by MHL in both strains, but the repression levels and timing of expression differed between genotypes). The enhanced induction of antioxidant enzyme genes exhibited by Δ*cor-5* in response to short and prolonged treatments with ABA or after short term exposure to MHL was consistent with the increased levels of ROS accumulation found in the mutant lines.

To gain insight into the mechanism by which lack of *PpCOR413im* prevented the ABA-induction of target genes, we compared the ABA-transcriptional activation of target genes, in WT, *Δcor-5* and another *P. patens* strain (*hxk1*) which exhibit hypersensitivity to ABA (Thelander et al., 2005). *hxk1* harbors a KO mutation in a type A hexokinase gene, a major chloroplast stromal- glucose phosphorylating enzyme that has been shown to be involved in light and energy-mediated growth responses (Olsson et al., 2003; Thelander et al., 2005). In addition to ABA hypersensitivity, the reported developmental alterations of *hxk1* share striking phenotypic similarities with those exhibited by *PpCOR413im* KO mutants. Moreover, growth of *hxk1* strain under MHL or HL intensities was largely repressed (Supplementary Figures 6A,B).

To compare the transcriptional responses between the different strains, colonies of WT, *Δcor-5* and *hxk1* were treated for 12 h with 10 μM ABA and RNA samples were analyzed by qRT-PCR for the expression of genes involved in ABA biosynthesis (*NCED*), ABA responses (*TSPO*), and ROS detoxification (*CAT*) (Supplementary Figure 6C). While *Δcor-5* showed a significant reduction in the ABA induction of *NCED* and *TSPO* genes (but not of *CAT*), *hxk1* mutant showed a complete inhibition of ABA-mediated gene expression, including CAT gene. Taken together, these results suggest that the mechanisms employed by PpCOR413im and HXK1 to regulate gene expression and responses to ABA, are at least partially overlapping.

### Disruption of *PpCOR413im* Resulted in Changes in the Chloroplast Ultrastructure Without Affecting the Photosynthetic Complexes in the Thylakoid Membranes

The predicted subcellular localization of PpCOR413 has led to the suggestion that this type of proteins may play a structural function by preserving membrane stability under stress. To gain insight into this hypothesis, we used electron microscopy to investigate whether disruption of *PpCOR413im* resulted in alterations in the ultrastructure of chloroplasts. WT and Δ*cor-5* colonies grown at standard light conditions or exposed for 48 h to HL, were fixed and processed for electron microscopic visualization.

**FIGURE 8:**
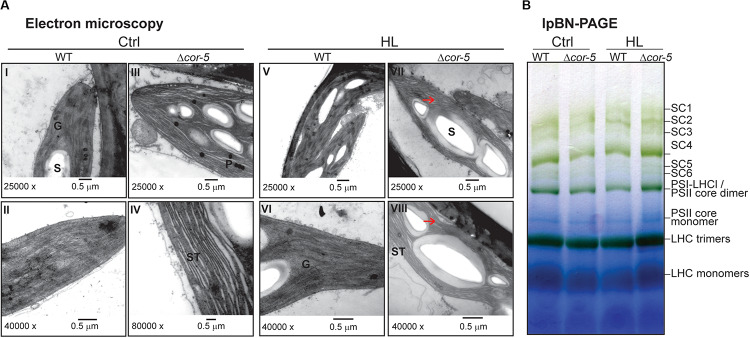
Comparison of chloroplast ultrastructure and thylakoid protein complexes WT or *PpCOR413im* KO strains. **(A)** Colonies of *P. patens* WT and Δ*cor-5* lines were grown under standard light and temperature conditions during 20 days and thereafter exposed to high light (HL: 350 μmol m^–2^ sec^–1^), during 48 h. Samples of gametophores were taken from controls (Ctrl) (I-IV) or after HL treatment (V-VIII) and processed for transmission electron microscopy. G: granum, S: starch, ST: stromal thylakoids. Unstructured thylakoids are indicated with arrowheads. Magnifications and scale bars are shown below each micrograph. **(B)** Large pore Blue Native-PAGE of thylakoid protein complexes from WT or *Δcor-5* strains, grown under standard light conditions (Ctrl) or exposed for 48 h to high light (HL: 350 μmol m^–2^ sec^–1^). Thylakoids were isolated from 20 days-old colonies (controls or treated), solubilized with 1% β-DM and proteins corresponding to 15 μg of chlorophyll per sample were loaded in the wells. The complexes in the samples are named according to Gerotto et al. (2019). The high molecular weight complexes in the lpBN-gels correspond to super-complexes (SC); SC1: PSII C2S2M2; SC2: PSII C2S2M; SC3: PSII C2S2; SC4: PSI-large; SC5: PSII C2S; SC6: PSI-LHCI-LHCII.

Chloroplasts of WT plants cultivated on standard light conditions had well-structured internal membrane system, with several grana thylakoids and few starch grains in the stroma (Figure 8A I,II). These differed from chloroplasts from Δ*cor-5* grown under identical conditions in that the later ones had several starch grains and few grana. In addition, stroma thylakoids from Δ*cor-5* chloroplasts exhibited a more relaxed and disorganized orientation (Figure 8A III, IV).

Exposure of plants to HL did not significantly affect the structure of the chloroplasts from WT plants, except for the presence of a larger number of starch grains (Figure 8A V, VI). In contrast, chloroplasts from KO plants had more and larger starch grains than chloroplasts from WT grown under HL, and thylakoids were unstructured (Figure 8A VII, VIII).

This apparent distortion of chloroplast ultrastructure observed in Δ*cor-5* is consistent with a structural role of PpCOR413im in chloroplast membrane stabilization. Based on this presumption, a plausible explanation for the dramatic growth inhibition of the mutant lines under high light conditions is that the observed structural alterations in chloroplasts could affect photosynthesis and energy status by altering the composition of chlorophyll–protein complexes in the thylakoid membranes. To investigate this hypothesis, thylakoid membranes (with equal amounts of chlorophyll) were isolated from control or HL treated colonies of WT and *Δcor-5* strains, solubilized in 1% n-dodecyl-β-D-maltoside (DM) and analyzed by large pore Blue Native gel electrophoresis (lpBN-PAGE) (Figure 8B). The results showed no qualitative differences in the organization of the photosynthetic protein complexes between WT and *Δcor-5* strains under control light or after 48 h exposure to HL. This indicates that the mutant hypersensitivity to HL is not a direct consequence of a defected photosynthesis caused by alterations in protein complexes in the electron transport chain. In turn, other physiological roles dependent on membrane stability should be explored for this type of protein.

## Discussion

*Physcomitrella patens* is considered a valuable non-seed plant model for studying gene function from an evolutionary perspective. Using this model system, this work provides the first direct genetic evidence for the involvement of a chloroplastic COR413 protein, in responses to ABA, light intensity and various other environmental stresses.

*Physcomitrella patens* contains a single chloroplastic COR413 encoding gene (*PpCOR413im*) and four other genes encoding PM variants (Figure 1), indicating that the structure of the *COR413* gene family has been preserved during plant evolution, with a larger number of genes for PM localized proteins than of genes for chloroplast variants (Breton et al., 2003; Okawa et al., 2008). The absence of *COR413* genes in green algae, the closest freshwater relatives of land plants, suggests that these genes originated anciently in plants as part of the necessary mechanisms that plants developed to face new environmental challenges during land colonization.

PpCOR413im was found to reside in chloroplasts when expressed as a fusion to GFP (Figure 2A). Experimental evidence concerning subcellular localization of COR413im proteins from *Arabidopsis* AtCOR413IM-1 and AtCOR413IM-2 proteins demonstrated that these proteins localize on the inner membrane of chloroplasts with the C-terminus of the protein facing the stroma (Okawa et al., 2008; Lundquist et al., 2017). In addition, these studies also showed that AtCOR413IM form oligomeric complexes in the chloroplast inner membrane. Based on the similarities in subcellular localization, sequence and predicted structures observed between PpCOR413im and the *Arabidopsis* orthologs, it is conceivable that these proteins have similar biochemical functions.

Like other *COR413* genes, *PpCOR413im* was efficiently induced by ABA treatment and by various abiotic stresses, including LT, salinity and osmotic stress (Figure 2B). In addition, we showed that *PpCOR413im* was upregulated in response to HL, which has not been previously reported for this type of genes. This is not surprising since responses to HL partially overlap with the responses triggered by other abiotic stress factors. For example, LT interferes with photosynthesis by limiting electron transport and carbon fixation rates (Huner et al., 1998). In mosses, exposure to HL often results in plant dehydration, as these plants fail to control water content in cells (Proctor et al., 2007). Therefore, it is conceivable that mosses could have higher level of overlapping between HL and dehydration-induced adaptive pathways than vascular plants.

Our results showed that targeted disruption of *PpCOR413im* resulted in pleiotropic alterations in growth, development, and responses to abiotic stresses (excess light, LT, dehydration and osmotic stress).

Regarding growth and development, the main features of *PpCOR413im* mutants were: reduced growth rate at standard light conditions, alterations in the development of caulonema, and hypersensitivity to ABA.

With regard to stress-related phenotypic alterations of *PpCOR413im* mutants, we found that mutant lines were affected in photosynthesis performance under all stress conditions assessed, particularly upon exposure to LT and dehydration, suggesting that *PpCOR413im* is necessary for proper adjustment of photosynthetic activities under changing environmental conditions (Figure 5). Nevertheless, the results also indicated that disruption of *PpCOR413im* had different effects on photosynthesis depending on the type of stress.

For example, LT stress was the only assessed condition in which mutant lines showed increased susceptibility of PSII to photoinhibition, as no differences in Fv/Fm values were observed between WT and mutant genotypes upon severe dehydration or exposure to HL (Table 1).

Another variable that showed differences between WT and mutant genotypes was the induction of NPQ. This parameter has been shown to be an important mechanism in *P. patens* responses to HL, salinity, LT and drought (Gerotto et al., 2011; Beike et al., 2015; Gao et al., 2016; Tan et al., 2017). In fact, regulation of NPQ has been suggested to be the main system for suppression of HL-induced ROS formation in chloroplasts of *P. patens* (Alboresi et al., 2010). However, we found that NPQ levels were only marginally affected in *PpCOR413im* mutants upon exposure to HL, although these plants accumulated higher levels of ROS (superoxide) than the WT under these conditions (Figure 6A). This suggests that PpCOR413im activity is necessary for protection of plants from HL-induced photo-oxidation via a mechanism that is at least partially independent of NPQ.

Nevertheless, induction of NPQ was highly compromised in KO mutants after severe dehydration, or to a lower extent after exposure to LT, and in all situations assessed, the reduced levels of NPQ induction were not accompanied with differences in the Chl *a*/*b* ratio suggesting that the antenna size of photosystems remained unaffected by disruption of *PpCOR413im* (Supplementary Table 2).

*Physcomitrella patens* has unique characteristics of NPQ activation, since it relies on the presence of LHCSR and PSBS proteins from the light-harvesting complex superfamily, which are typical from both algae and vascular plants, respectively (Dominici et al., 2002; Peers et al., 2009; Alboresi et al., 2010; Gerotto et al., 2011; Pinnola et al., 2015). Remarkably, our results showed that the expression profiles of *LhcsR* or *PsbS* genes were not altered by *PpCOR413im* KO mutation, suggesting that lower NPQ levels of KO mutants are not directly associated with the misregulation of these genes at the transcriptional level (Supplementary Figure 5).

Despite the alterations in photochemical parameters observed under different stress stimuli, the most striking differences between the KO mutant lines and the WT, was the hypersensitivity to excess light intensities, manifested as a significant reduction of plant growth, particularly dramatic upon exposure to light intensities of 200 μmol m^–2^ sec^–1^ and above (Figure 3). To our knowledge, this is the first report describing a functional role for COR413 genes in the regulation of plant growth in response to changing light intensities.

In addition to surviving, plants must have the ability to adjust their growth and development to the existing environmental conditions. Abiotic stress perturbs the photosynthetic balance between the energy supplied and the energy consumed and therefore often induces the inhibition of plant growth (Gray et al., 1997; Miura and Furumoto, 2013; Pinnola et al., 2013). In *P. patens*, light intensity has been shown to play an important role regulating growth and development (Thelander et al., 2005). For example, energy supply regulates the balance between the two types of protonemal filaments, caulonema and chloronema. High energy, provided by glucose administration or exposure to light intensities ≥ 30 μmol m^–2^ sec^–1^, induce caulonema formation. Conversely, lower light intensities inhibit caulonemal growth and stimulate chloronemal branching (Thelander et al., 2005). Interestingly, KO mutants of *PpCOR413im* exhibited reduced caulonemal growth and increased chloronemal side branching when grown under standard light conditions (50 μmol m^–2^ sec^–1^) (Figure 4A). However, mutants were not impaired in caulonemal formation induced by auxin treatment or ammonium deprivation (Figure 4C), indicating that the ability to promote caulonemal growth and differentiation is retained in these strains. These phenotypic alterations very much resembled the reported phenotype of *hxk1*, a *P. patens* strain harboring a KO mutation of a gene encoding a chloroplast hexokinase gene encoding a type A hexokinase, a major chloroplast targeted glucose-phosphorylating enzyme (Olsson et al., 2003; Thelander et al., 2005). It was proposed that disruption of *PpHxk1* created a condition of artificial starvation as a result of a reduction in hexose phosphorylation and the consequent decrease of ATP production. Interestingly, we showed that exposure of *hxk1* to HL completely inhibited plant growth (Supplementary Figures 6A,B). Based on the similarities of *PpCOR413im* KO mutants with *hxk1*, it is tempting to hypothesize that deletion of *PpCOR413im* resulted in changes in energy and metabolism of plant cells, suggesting that PpCOR413im may be involved in stress-induced metabolic reprogramming. This is supported by the fact that *PpCOR413im* mutant strains exhibited similar growth rates than the WT when grown at low light conditions (10 μmol m^–2^ sec^–1^) (Figure 3B) or when the culture media was supplemented with glucose (Figure 4C). In fact, the phenotype of KO lines growing at standard growth conditions resembles the phenotype of WT or KO plants growing at low light. This suggests that under standard light conditions, KO plants exhibit growth and developmental features that are according to a low energy growth mode. Following this line of reasoning, it is likely that loss of *PpCOR413im* potentiated stress-induced energy imbalance leading to growth and developmental adjustment responses.

ABA is one of the important phytohormones controlling growth and development in *P. patens* (Komatsu et al., 2013; Zhao et al., 2018) and plays a key role in the regulation of chloroplast physiology during stress. A recent report demonstrated that during cold acclimation, Δ*abi3* mutants had reduced photosynthetic capacity, increased NPQ levels and alterations in the composition of photosynthetic protein complexes (Tan et al., 2017). Based on these results, the authors suggested that maintenance of PSII activity via ABA signaling pathway contributes to overcome the stress-induced energy imbalance during cold acclimation (Tan et al., 2017).

Our results showed that exposure to HL induced a moderate increase in ABA levels in Δ*cor-5* but not in the WT (Figure 4B). Since this condition induced higher dehydration levels in KO plants than in the WT, it cannot be ruled out the possibility that ABA accumulation was due to increased water loss. These results also weight the importance of *PpCOR413im* in responses to dehydration, since mutants were clearly affected in photochemical parameters under this stress condition. This function could be particularly relevant for poikilohydric plants, such as mosses, where photosynthesis is highly dependent on water availability.

In contrast to the reported LT-induced ABA accumulation in *P. patens* (Beike et al., 2015), we did not detect elevated levels of endogenous ABA in WT or Δ*cor-5* after 48 h exposure to LT. The discrepancies in the reported ABA levels may be explained by differences in the experimental conditions employed in the studies. In fact, our data is consistent with results from Minami et al., 2005, showing no increase in ABA in response to cold.

Our results showed that *PpCOR413im*-defective mutants were hypersensitive to ABA as seen by the exacerbated shape of protonemal cells and the enhanced growth inhibition in response to ABA treatment (Figure 4C). This phenotype was remarkably similar to the reported ABA hypersensitivity of *hxk1* mutant (Thelander et al., 2005), and opposite to the growth and developmental alterations observed in *Δabi3* (Marella et al., 2006; Zhao et al., 2018). In contrast to Δ*cor-5* and *hxk1*, *Δabi3* showed accelerated caulonema formation and reduced chloronema branching (Zhao et al., 2018). Taking together, these results support the notion that disruption of *PpCOR413im* interferes with ABA-induced developmental responses.

In *Arabidopsis*, mutations in some genes involved in the regulation of *COR* gene expression, have been shown to exhibit ABA hypersensitive phenotypes (Perruc et al., 2007; Belin and Lopez-Molina, 2008; Kang et al., 2018). An example of such a regulatory gene is *PKL*, a putative CHD3-type chromatin remodeling factor with important roles in plant growth and development and responses to abiotic stress (Yang et al., 2017, 2019). This indicates that, at least some COR proteins have regulatory roles in ABA-dependent responses.

Further evidence supporting a role for *PpCOR413im* in ABA responses was provided by the observed alterations in Δ*cor-5* expression profiles of genes involved in ABA synthesis and signaling, as well as stress responsive genes, including genes encoding antioxidant enzymes. For most genes, the induction levels reached after ABA treatment were significantly lower in Δ*cor-5* than in the WT, particularly after short-term treatments (Figure 7). These results are consistent with a role for *PpCOR413im* in events occurring downstream of ABA production and suggest that PpCOR43im plays a direct or indirect role in the regulation of nuclear gene expression. Interestingly, in contrast to the WT, Δ*cor-5* failed to induce the ABA biosynthetic gene *NCED* in response to HL, which contradicts the increased ABA levels found in the mutant under these conditions. However, the expression of *PP2C*, a gene involved in inhibition of ABA signaling (Ma et al., 2009; Park et al., 2009; Komatsu et al., 2013), was induced in the WT but not in Δ*cor-5* after 72 h treatment with HL, suggesting that mutant lines may be affected in the ability to repress ABA signaling after prolonged exposure to stress. Similarly, Δ*cor-5* plants were unable to trigger and/or sustain stress-related gene expression after long-term exposure to ABA or HL. Interestingly, *PpCOR413im* defective mutants exhibited higher levels of *CAT* transcript accumulation than the WT in response to all treatments, but failed in the long-term induction of *SOD* and *PRX*. These two genes encode chloroplastic enzymes, suggesting that loss of *PpCOR413im* interferes with the ability to detoxify ROS within this organelle. The higher levels of stress-induced ROS accumulation detected in *PpCOR413im*-defective lines supports this observation.

Our results showed that expression of ABA-responsive genes was predominantly inhibited in *PpCOR413im* disrupted lines, evidencing an apparent inconsistency with the ABA hypersensitivity of the KO mutants. Nevertheless, analysis of the transcriptional response to ABA in the *hxk1* mutant, which has been previously reported to be hypersensitive to ABA (Thelander et al., 2005), showed that *hxk1* also failed to induce gene expression in response to exogenously added ABA (Supplementary Figure 6C). In fact, disruption of *hxk1* resulted in a complete inhibition of ABA-mediated gene expression, including CAT gene. Taken together these results suggest that PpCOR413im and HXK1 regulate responses to ABA via partially overlapping mechanisms, and that these mechanisms probably depend on sensing of cellular energy status.

Besides the effects on nuclear gene expression, disruption of *PpCOR413im* resulted in the alteration of chloroplast gene expression. Our results showed that in WT plants, exposure to HL induced *PsbA* and *PsbD* gene expression and that ABA down-regulated *PsbD*. However, in KO mutants, *PsbA* and *PsbD* gene expression reached similar expression levels under all conditions assessed, suggesting that ABA-mediated transcriptional responses of chloroplastic genes were also altered by the loss of *PpCOR413im*.

Taking into account that both nuclear and chloroplast gene expression were affected in the KO mutants, it is possible that *PpCOR413im* plays a role in chloroplast retrograde signaling to regulate the expression of stress-related genes. Following this line of reasoning, *PpCOR413im* may play a role in the accumulation of specific compounds in chloroplasts which may in turn, act as retrograde signals (Estavillo et al., 2011; Xiao et al., 2013; Norén et al., 2016; Zhu, 2016).

This hypothesis is supported by the knowledge that ABA contributes to stress-induced metabolic reprogramming in *P. patens*, by regulating the accumulation of a large number of metabolites, including sugars, amino acids and organic acids (Arif et al., 2018).

Concerning sugar metabolites, *P. patens* suffers important changes in starch and soluble sugar content under a variety of abiotic stresses and in response to ABA (Bhyan et al., 2012; Takezawa et al., 2015; Gao et al., 2016; Arif et al., 2018). Sucrose is the main product of photosynthesis and a dominant regulator of plant growth. When energy supply is limited, chloroplast-stored starch and sucrose can be used for providing energy and metabolites to sustain metabolism and growth (Smith et al., 2005; Fettke et al., 2009). Thus, stress adaptation requires a proper control of sugar metabolism and compartmentation.

Our results showed that chloroplasts of *PpCOR413*-disrupted plants accumulated more starch than the WT upon exposure to HL, and exhibited higher levels of sucrose in HL- or ABA-treated plants (Figures 6C,D). This result was similar to the observed increases in starch content of *P. patens* lines harboring KO mutations in *Lhcb5* or *Lhcb6*, and showing reduced NPQ activation in response to HL (Peng et al., 2019). However, in contrast to *Lhcb5* and *Lhcb6 KO* lines, disruption of *PpCOR413im* did not result in a major reduction of NPQ induction by HL, although significant decreases in NPQ levels were observed after dehydration or LT treatments. Therefore, regarding the responses to HL, it is unlikely that the alterations in starch accumulation of *PpCOR413im* KO lines occurred as a consequence of a reduced ability to induce NPQ of the mutants. Thus, further experiments are required to determine whether these phenomena are indirectly linked to other physiological processes that could have been affected by this mutation and by mutations of *Lhcb5* and *Lhcb6*.

A possible explanation for the observed changes in sugar content is that loss of *PpCOR413im* could alter the intracellular distribution of metabolites having roles in chloroplast physiology. This hypothesis is supported by a recent study showing that *Arabidopsis* KO mutants of a gene encoding a predicted PM targeted COR413 protein (COR413-PM1), showed significant alterations in the accumulation of proteins involved in the metabolisms of fatty acids, fructose, starch and sucrose (Su et al., 2018). Based on these results, it was suggested that the *Arabidopsis* COR413-PM1 regulates plant tolerance to freezing stress by affecting different metabolic pathways. Moreover, the structural features of COR413 proteins are compatible with those displayed by membrane transporters (Okawa et al., 2008). There is enough evidence showing that different metabolites are transported across membranes via transporter proteins that play a role in plant stress responses (Stitt and Hurry, 2002; Lundmark et al., 2006; Guy et al., 2008; Roston et al., 2012; Patzke et al., 2019). In plants, the intracellular and intercellular distribution of sugars depends on specific sugar transporters that reside in the plasma membrane or in membranes of different organelles (Bush, 1999; Chen et al., 2015; Hedrich et al., 2015; Patzke et al., 2019). A number of sugar transporters have been identified in chloroplasts, including the maltose exporter MEX1 (Niittylä et al., 2004; Findinier et al., 2017), the plastidic glucose transporter pGlcT (Weber et al., 2000) and the plastidic sugar transporter (pSuT) (Patzke et al., 2019). The analysis of KO mutants altered in sugar transporter genes has highlighted the importance of these genes in developmental and stress responses in plants. For example, disruption of *MEX1* resulted in accumulation of maltose in chloroplast stroma, inhibition of starch degradation and growth retardation (Niittylä et al., 2004). Moreover, an *Arabidopsis* mutant defective in *pSuT*, was affected in glucose and sucrose export out of chloroplasts and exhibited altered developmental responses and responses to cold (Patzke et al., 2019).

Similar to the *Arabidopsis* COR413im proteins many chloroplast metabolite transporters characterized to date reside in the inner envelope. Therefore, it is possible that COR413im proteins are involved in stress-induced metabolic adjustment by acting as transporters or interacting physically or functionally with other metabolite transporters to regulate their activity. To date, the only candidate protein that has been suggested to interact with COR413im is RETICULATA (RE), a protein of unknown function that is also targeted to the chloroplast inner membrane (Lundquist et al., 2017). Although the function of RE remains unknown, KO mutants of this gene exhibit alterations in lipid and amino acid metabolites and are affected in developmental processes (Lundquist et al., 2014, 2017).

Alternatively, PpCOR413im may play a structural role by stabilizing the chloroplast inner membrane during stress, as suggested for other COR413 proteins (Okawa et al., 2008; Zhu, 2016). The fact that chloroplasts of *PpCOR413im* defective mutants showed important ultrastructural changes in the organization of the internal membrane system (Figure 8A), supports this hypothesis. However, these changes did not affect the organization of chlorophyll–protein complexes in the thylakoid membranes (Figure 8B). Therefore, it is unlikely that the structural role of PpCOR413im involves stabilizing the photosynthetic protein complexes on the thylakoid membranes, but rather act on other membrane proteins such as transporters, receptors or channels.

Moreover, in other moss species, ABA treatment has been linked with improved membrane stability during desiccation (Werner et al., 1991; Bopp and Werner, 1993; Beckett, 1999; Guschina et al., 2002; Khandelwal et al., 2010; Pressel and Duckett, 2010; Stevenson et al., 2016; Tan et al., 2017). Therefore, it is possible that the ABA-induced accumulation of COR413 proteins improve membrane stability thereby allowing other integral membrane proteins to exert their function during stress.

In summary, we showed that a chloroplast resident COR413 protein from *P. patens* plays an important role in adaptive responses to various environmental stresses, including HL, dehydration and cold. Moreover, this study extends our understanding of the function of these plant-specific proteins in developmental and growth responses, and suggests that *PpCOR413im* participate in the regulation of these processes by coordinating cellular energy status information with signaling events derived from ABA and abiotic stress conditions.

Understanding the precise biological function of PpCOR413im by reverse genetics is challenging because it requires the dissociation of the mechanisms underlying the different pleiotropic effects observed in the disruption mutants. Nevertheless, we present a model suggesting that PpCOR413im acts downstream of ABA and contributes to chloroplast homeostasis and metabolic adjustment under challenging environmental conditions. Although our data strongly suggest that PpCOR413im is required for maintaining membrane properties under environmental stress, with the current knowledge is not possible to determine whether this protein exerts its function directly or indirectly via interaction with other membrane integral proteins. Further studies are required to understand the biochemical function of PpCOR413im and other COR413 proteins.

## Data Availability Statement

The datasets generated for this study are available on request to the corresponding author.

## Author Contributions

SV and CR: conceive and design the experiments. Performed the experiments: CR (generation, phenotypical and molecular characterization of KO lines), AC (expression analysis), AF and GQ (photochemical analysis), JQ (participated in construct generation and plant transformation), SV, CR, AC, and AF (analyzed the data), SV (wrote the manuscript). All authors contributed to the article and approved the submitted version.

## Conflict of Interest

The authors declare that the research was conducted in the absence of any commercial or financial relationships that could be construed as a potential conflict of interest.

## Footnotes

1 https://genevestigator.com/

2 http://bar.utoronto.ca/efp_physcomitrella/cgi-bin/efpWeb.cgi
