# Supplementary material for: A Chloroplast COR413 Protein From Physcomitrella patens Is Required for Growth Regulation Under High Light and ABA Responses
Source: Front Plant Sci. 2020 Jun 19;11:845. doi: 10.3389/fpls.2020.00845 (PMC7317016; doi:10.3389/fpls.2020.00845)
Supplement: Supplementary file 1 [file Data_Sheet_1.PDF]

Supplementary Material

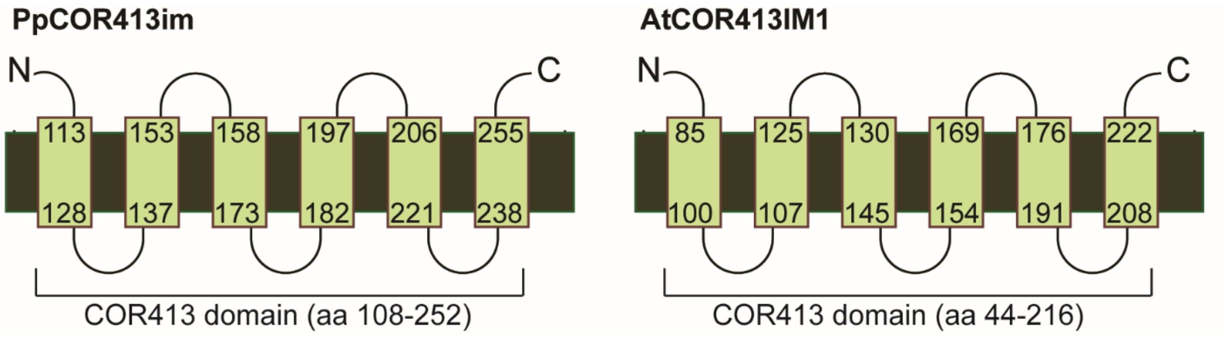

Supplementary Figure 1. Predicted topology of PpCOR413im and Arabidopsis AtCOR413IM1. Numbers in green rectangles represent amino acid positions at the beginning and at the end end of each predicted transmembrane domain. Region comprising COR413 domains are marked below. Amino acid (aa) positions at the beginning and at the end of the predicted conserved COR413 domain are shown.

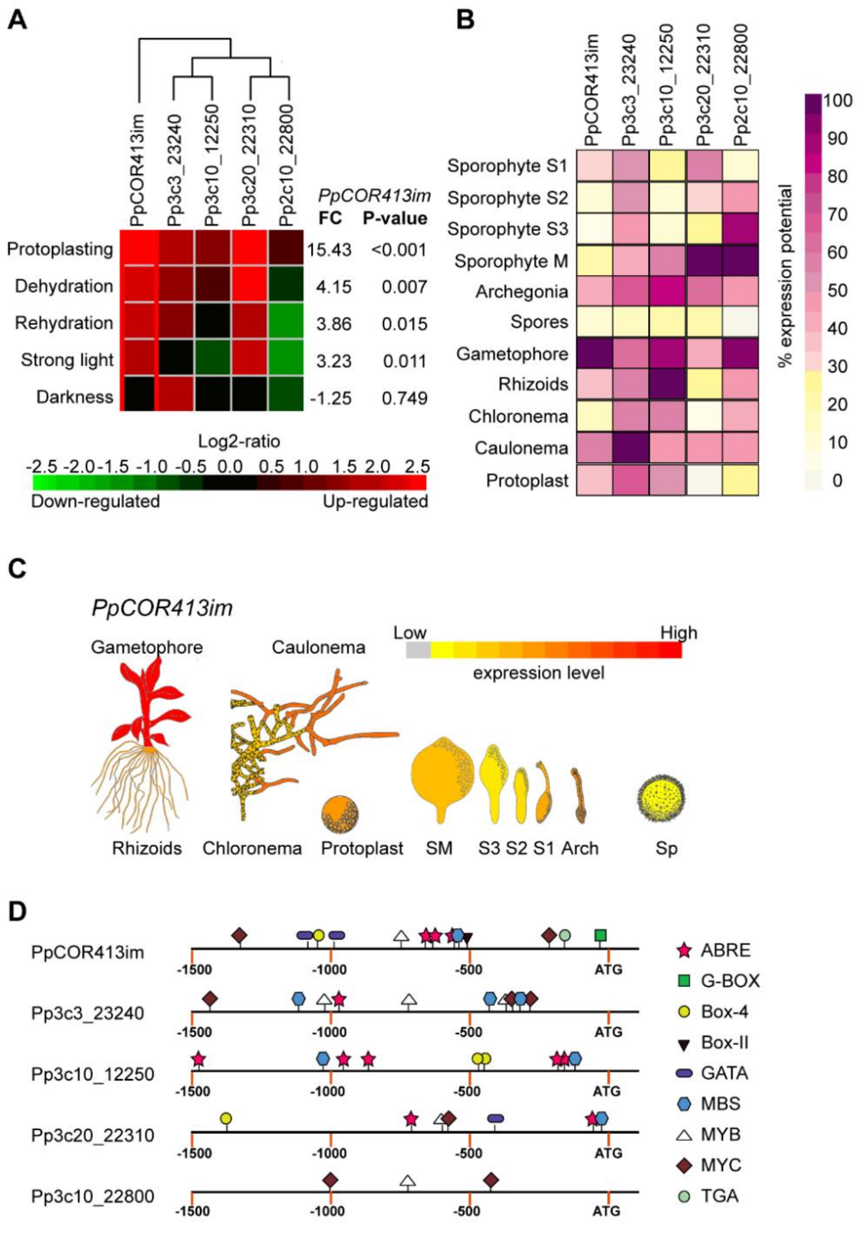

Supplementary Figure 2. *In silico* gene expression of *P. patens COR413 genes* during development and after different perturbations. **(A)** Comparison of gene expression of *P. patens COR413* genes using the Perturbation tool to search the microarray data of Genevestigator. To the right, fold-changes (FC) and P-values for *PpCOR413im gene*. Five perturbations in which PpCOR413im was specifically upregulated, are shown. The Gene ID and phylogenetic relationships between them are shown above the expression matrix. Upregulation is shown in red and downregulation in green. **(B)** Expression levels of *P. patens COR413* gene family during different developmental stages (eFP Browser data). **(C)** Developmental map of *PpCOR413im* gene expression (eFP Browser data). **(D)** *In silico* analysis of cis-regulatory elements present in COR413 promoters from *P. patens*. Promoter sequences from *P. patens COR413* genes (1500 bp upstream of the translation initiation site) were retrieved from Phytozome database and analyzed with PLACE, PlantCARE and PlantPAN 3.0 tools. Cis-regulatory elements are shown above of each promoter region, and distance (in bp) to the start codon (ATG) is marked below.

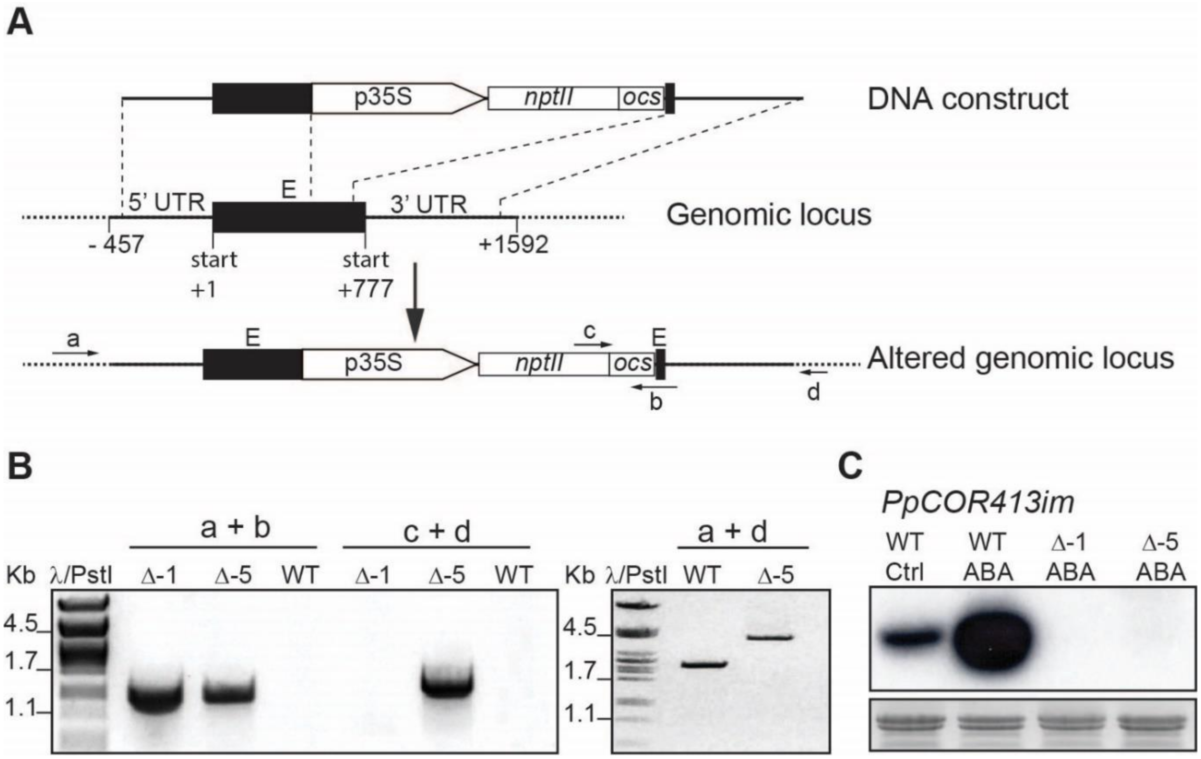

Supplementary Figure 3. Generation and molecular characterization of *PpCOR413im* disruption mutants. **(A)** Schematic diagram of targeting construct, *PpCOR413im* genomic locus and predicted targeted genomic locus. E: exon; 5' UTR (5' untranslated region); 3' UTR (3' untranslated region); p35S: cauliflower mosaic virus 35S promoter; *nptII*: kanamyicin resistance gene; *ocs* : octopine synthase terminator. The genomic nucleotide positions, present in the targeting construct, are shown are shown below in the genomic locus diagram. Primers used for identification of recombinant events are shown in the altered genomic locus diagram. **(B)** PCR genotyping analysis of wild-type (WT), Δ*cor-1* (Δ-1) and Δ*cor-5* (Δ-5) strains. PCR amplification and electrophoresis on agarose gels were performed from genomic DNA using the combination of primers shown in the figure. λ/PstI DNA molecular weight marker is shown to the left of each figure. The expected sizes of the PCR products that should originate from specific 5' or 3' homologous recombination (HR) events at the *PpCOR413im* locus are of 803 bp or 771 bp, respectively. For true gene replacement events, PCR using primer combination (a + d) should lead to amplification products of 2230 bp in WT and of 3928 bp. **(C)** Transcript accumulation of *PpCOR413im* in WT , Δ*cor-1* (Δ-1) and Δ*cor-5* (Δ-5) mutant lines. Total RNA was isolated from plants treated with 50 µM ABA for 24 hours or WT controls and analyzed by Northern blot using the full-length cDNA sequence of *PpCOR413im*, labeled with [α^{32}P]-dCTP as a probe. Ethidium bromide staining of rRNA was used to ensure equal loading of RNA samples.

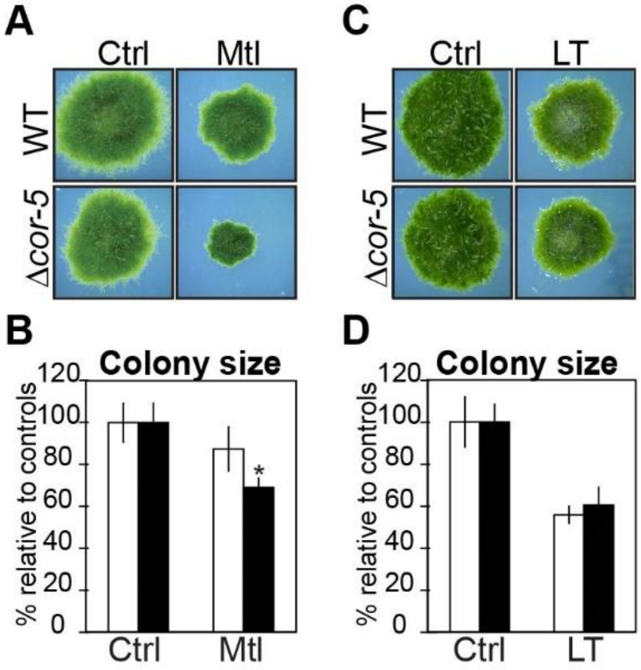

Supplementary Figure 4. Growth of WT and *Δcor-5* under osmotic stress or low temperature conditions. **(A)** Colonies from WT or *Δcor-5* were grown under standard light conditions (50 µmol m^{-2} sec^{-1}), in 200 mM mannitol supplemented plates (Mtl), or in regular growth medium (Ctrl). Photographic images were taken after 20 days and pictures of representative colonies from WT and *Δcor-5* are shown. **(B)** The area of 32 colonies grown as in A, was quantified using ImageJ. Colony size is expressed as a percentage of the values obtained from each genotype grown under optimal conditions, which were set as 100%. **(C)** Plants were grown for 15 days at standard light and temperature conditions, and thereafter transferred to a low temperature (LT) conditions (0-2 °C) for 10 days. Controls (Ctrl) were kept continuously at standard temperature conditions. Pictures of representative colonies from each genotype are shown. **(D)** The area of 32 colonies grown as in C, was quantified using ImageJ. Colony size is expressed as a percentage of the values obtained from each genotype grown under optimal conditions, which were set as 100%. In all cases, the values shown are means from one representative technical replicate. Error bars indicate SD (n = 32). Three biological replicates were carried out. Significant differences of at least 0.05 confidence level between the WT and the KO lines are marked with an asterisk.

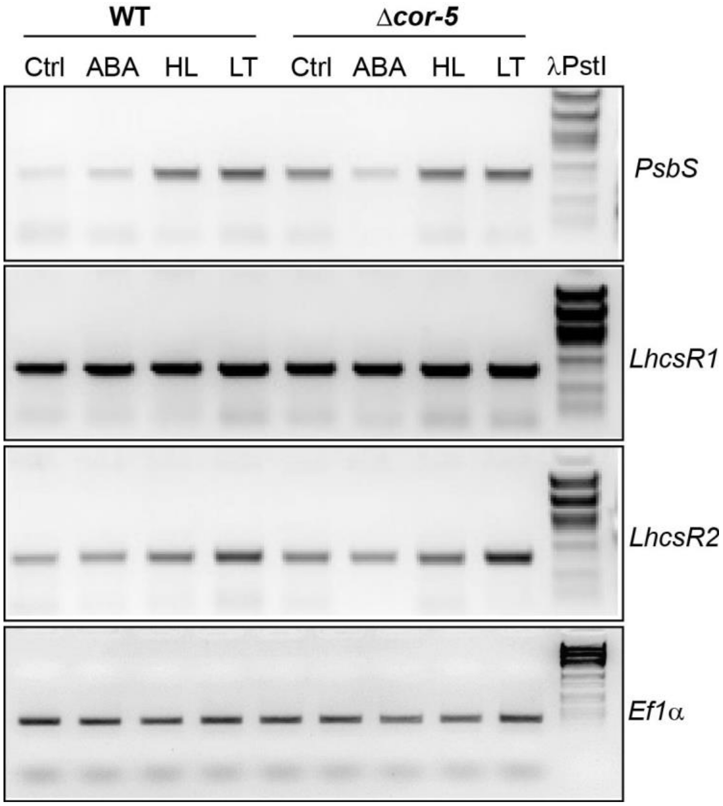

Supplementary Figure 5. Expression of *LHCSR* and *PSBS* genes from *P. patens* WT and *Δcor-5* strains in response to high light, low temperature and ABA treatments. Total RNA was isolated from plants treated with 10 µM ABA, or incubated with high light intensities (HL: 350 µmol.m^{-2}.sec^{-1}) or low temperature (LT: 0-2 °C) for 24 hours. Two micrograms of total RNA were reverse transcribed and analyzed by semi-quantitative RT-PCR analysis. *Elongation factor 1α* gene (Pp3c2_6770) was used as the reference gene to ensure equal cDNA concentrations in the reactions. Primers for Ef-1α amplification were Fw: 5´-tttgggattgaaatgtcgtg-3´ and Rev: 5´- tgagcatgagaaattgggtct-3´; for *PsbS* gene (Pp3c20_23430) were Fw: 5´-acccctcatcttgttcaacg-3´ and Rev: 5´-tgtgtcgtgctcggttagag-3´; for *LhcsR1* gene (Pp3c9_3440), were Fw: 5´-attgtgcgatgtctccatga-3´ and Rev: 5´-tttggaacgggtttagatcg-3´ and for *LhcsR2* gene (Pp3c15_11070) were Fw: 5´-accaaggagctcaacaatgg-3´ and Rev: 5´-gcctccggcattaaaataca-3´.

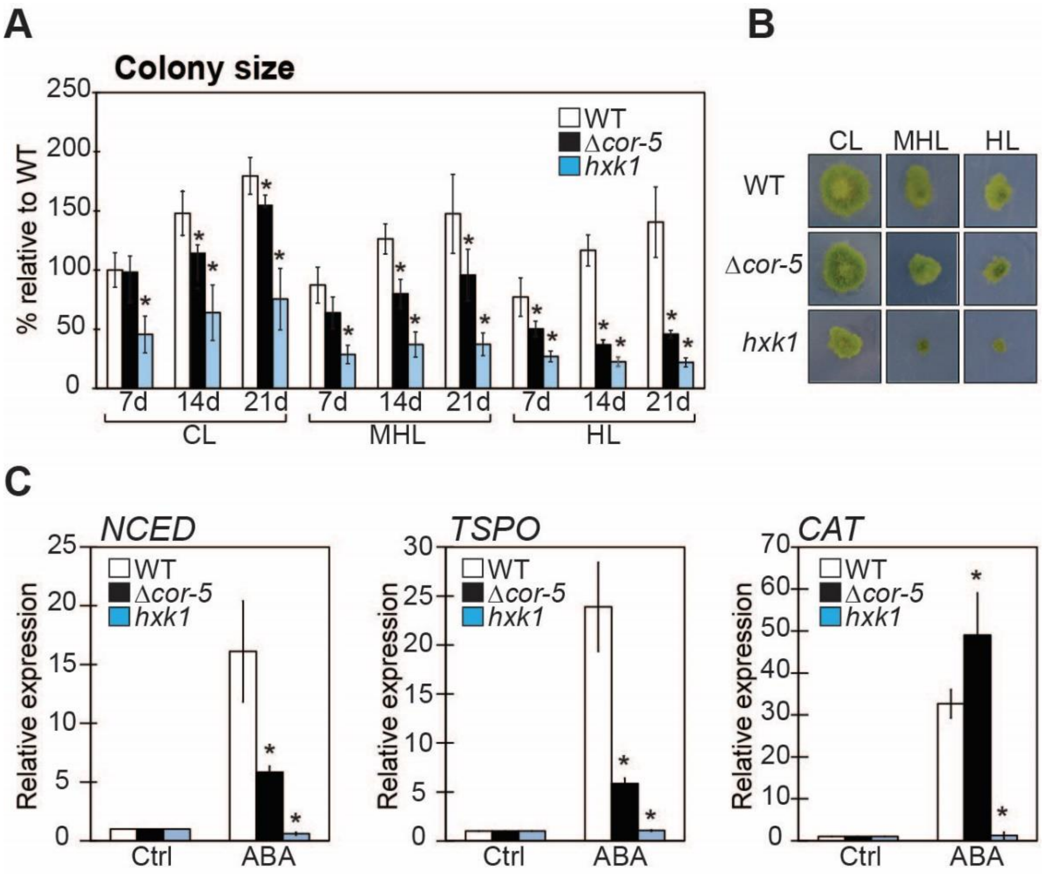

Supplementary Figure 6. Comparison of growth and gene expression between WT, *Δcor*-5 and *hxk1* strains. **(A)** Colonies of WT, *Δcor*-5 and *hxk1* genotypes were grown during 21 days at standard light conditions (CL: 50 µmol m^{-2} sec^{-1}), medium high light (MHL: 200 µmol m^{-2} sec^{-1}), or high light (HL: 350 µmol m^{-2} sec^{-1}). Photographic images were taken after 7, 14 or 21 days (d), and the area of 32 colonies was quantified using ImageJ. Colony size is expressed as a percentage of the value obtained from the WT genotype after 7 days of growth at standard light conditions, which was set as 100 %. Values are means from one representative technical replicate. Error bars indicate SD (n = 32). Two biological replicates were carried out. Significant differences of at least 0.05 confidence level between the WT and the KO lines are marked with an asterisk. **(B)** Pictures of representative colonies grown during 21 days as described in A. **(C)** Comparison of the expression levels of genes involved in ABA biosynthesis, stress responses and detoxification in WT, *Δcor-5* and *hxk1* strains. Twenty days-old colonies of each genotype were treated with ABA (10 µM) for 12 h and total RNA samples were isolated from treated (ABA) or untreated controls (Ctrl) for the analysis of *NCED* (Pp3c16_17210), *TSPO* (Pp3c2_17540) and *CAT* (Pp3c19_6540) gene expression by quantitative RT-PCR. The *Elongation factor 1α* gene (Pp3c2_6770) was used as internal control. Expression levels are reported as relative to control samples, and values represent means ± SE of 3 biological replicates. Significant differences of at least 0.05 confidence level between the WT and the mutant strains are marked with an asterisk. Specific primers used for PCR amplification are shown in Supplementary Table 3.

Supplementary Table 1. Predicted intracellular localization of *P. patens* COR413 protein family.

Acc. number/name	Localization and score	Signal type and score
Pp3c7_22090/PpCOR413im	**CHL** (0.663)	**cTP** (0.832)
Pp3c20_22310	**SEC** (0.657)	-
Pp3c10_22800	**SEC** (0.775)	-
Pp3c10_12250	**SEC** (0.673)	-
Pp3c3_23240	**SEC** (0.466)	-

Subcellular localization was predicted with GTP-Pp, a prediction tool trained with sequences from *Physcomitrella patens* (Fuss et al 2013). TargetP-2.0 was used to predict the presence of targeting signals. **CHL**: chloroplast; **SEC**: secreted; **cTP** (chloroplast Transit Peptide).

Supplementary Table 2. Chl *a/b*

Strain	Ctrl	HL	LT
WT	2.577 ± 0.455	1.979 ± 0.152	2.637 ± 0.267
Δcor-5	2.244 ± 0.237	2.072 ± 0.233	2.493 ± 0.329

Chlorophyll *a/b* ratio was evaluated in wild type (WT) and *Δcor-5* colonies grown under standard growth conditions (Ctrl: 24°C and 50 µmoles m-2 s-1), or after 24 h exposure to high light (HL: 24°C and 350 µmoles m-2 s-1) or low temperature (LT: 0-2°C and 50 µmoles m-2 s-1). Values are reported as mean ± standard deviation (*n* = 10). The values show no significant differences between WT and *Δcor-5*.

Supplementary Table 3. Primers used for qRT-PCR

Gene name	Abbr	Acc. No.	Primer Forward	Primer Reverse
Protein phosphatase 2C	PP2C	Pp3c14_25570	tgtgcctatacgtggaccaa	cgcttgcaggtcttgaggttc
9-cis-epoxycarotenoid dioxygenase	NCED	Pp3c16_17210	ccgtgaagttcaagcactga	gtatcctccaccacgctgtt
Sucrose synthase	SUS	Pp3c5_19770	cacaccagccgatgtacaag	ccgctaatcccatcagaaac
Translocator protein	TSPO	Pp3c2_17540	cgtggttagtgtgggctga	agtaagcaccgagagcgaaa
Superoxide dismutase	SOD	Pp3c9_24840	tggaagggcattcgttattc	tggagggcattcgttattc
Dehydrin A	DHNA	Pp3c12_900	cctgagactatggtaggcgg	caaatccttttcccctcctc
Catalase	CAT	Pp3c19_6540	tgcgaaataggatgtgcgta	catgaatgaaatggggaagg
Class III peroxidase	PRX	Pp3c4_14530	caatacgctactcgcgactctgt	cgtctcttcgaccgccata
Elongation factor 1α	EF1a	Pp3c2_6770	aggcagacgtgggttacttga	cattaatcatcaggcaggcata
